# Vibrational spectroscopy methodology for profiling of mixed carbon metabolism in carotenogenic and oleaginous *Mucor circinelloides*

**DOI:** 10.1186/s12934-026-02963-6

**Published:** 2026-02-21

**Authors:** Simona Dzurendová, Eirik Almklov Magnussen, Volha Shapaval, Achim Kohler, Boris Zimmermann

**Affiliations:** 1 Faculty of Science and Technology, Norwegian University of Life Sciences, Drøbakveien 31, P.O. Box 5003, Ås, 1432 Norway; 2 Faculty of Chemistry, Brno University of Technology, Brno, Czechia Czechia

**Keywords:** Oleochemicals, Biodiesel, Microbial biopolymers, Fourier transform infrared spectroscopy, Fourier transform Raman spectroscopy, Microspectroscopy, Mucor

## Abstract

**Background:**

Mixed carbon substrate fermentation is gaining interest in industrial biotechnology for effectively utilizing sustainable resources like waste glycerol and lignocellulose to produce food, feed, biofuels, and platform chemicals. However, challenges arise from the inefficient co-utilization of carbon sources as most microorganisms exhibit a preference for one substrate. This study evaluated the use of stable isotope labelling with infrared and Raman spectroscopies and microspectroscopies to investigate carbon utilization in fermentations with mixed carbon substrates. As a model system, the carotenogenic and oleaginous filamentous fungus *Mucor circinelloides* was grown on varying ratios of glucose and glycerol under nitrogen-limited conditions to induce lipid accumulation during the stationary growth phase.

**Results:**

The multi-modal spectroscopic approach successfully identified the flux of carbon from mixed substrates (glucose and glycerol) into specific metabolites, providing a detailed timeline of metabolite production. In the early phase of fermentation (first 8 h), biomass was rich in proteins and carbohydrates, primarily resulting from yeast extract utilization and some glucose consumption. Between 8 and 14 h, the production of polyphosphates, lipids, and carotenoids began. In media with abundant glycerol, carotenoids were assembled from both glucose and glycerol, with potential contributions from yeast extract. The lipid accumulation, primarily in the form of triglycerides (TAGs), is largely attributed to the utilization of glucose. Beyond 14 h, the biomass continues to accumulate polyphosphates, while high TAG levels were only observed when glucose was plentiful. In contrast, media with excess glycerol or glycerol as the sole carbon source resulted in only modest or negligible TAG accumulation, respectively. Polyphosphates were identified as important additional energy reserves alongside TAGs.

**Conclusions:**

This study demonstrates that stable isotope labelling coupled with multi-modal infrared and Raman spectroscopies is a powerful approach for tracking carbon flow in mixed substrate fermentations. The findings highlight the pivotal roles of glucose in lipid accumulation and polyphosphates as alternative energy reserves in *Mucor circinelloides*. The combined use of multiple infrared and Raman techniques revealed complementary spectral features, improving data reliability and providing a comprehensive chemical insight into the fermentation process. This approach can contribute to the development of more efficient bioprocesses for sustainable production in industrial biotechnology.

**Supplementary Information:**

The online version contains supplementary material available at 10.1186/s12934-026-02963-6.

## Introduction

Fermentation processes based on mixed carbon substrates, where a microorganism grows on two or more carbon sources, such as carbohydrates, alcohols and organic acids, are gaining increased interest in industrial biotechnology [1]. In particular, microscopic fungi can metabolize a wide range of organic compounds which makes them ideal for the production of food, feed, biofuels and platform chemicals by utilisation of various inexpensive carbon-rich substrates sourced from waste, side-streams and rest biomass [2]. Waste glycerol from biodiesel production and fish oil refinery and lignocellulose biomass from agricultural and forestry waste are examples of environmentally friendly and cost-effective carbon sources for microbial biotechnology [3–5]. Furthermore, lignocellulosic hydrolysates consist of a complex mixture of various carbohydrates and short-chain aliphatic acids, with glucose and xylose as the predominant components [6]. Carbon sources, such as glucose and xylose, are metabolized by fungi at different rates due to variations in transport mechanisms, enzymatic affinities, and regulatory pathways, often resulting in sequential or preferential substrate (usually glucose) utilization, also known as carbon catabolite repression Therefore, understanding co-utilization of mixed carbon substrates during fungal fermentation is of high importance. However, a fermentation approach based on mixed carbon substrate has a number of challenges, primarily related to efficient co-utilisation of all carbon substrates. Most organisms have clear preference towards one of the carbon sources, leading to sequential or selective consumption of substrates, thus prolonging the process and reducing the efficiency of carbon utilisation [7, 8]. This problem can be tackled by either metabolic engineering strategies via modification of metabolic pathways, or nutrient management strategies via adjusting the dynamic concentration profiles of each carbon source.

Usually, high-performance liquid chromatography (HPLC) and gas chromatography (GC) are used to trace the consumption of different carbon sources and their incorporation into microbial metabolites. However, the chromatography methods are often costly and time-consuming since they require substantial amount of sample, multi-step extraction and preprocessing steps. Vibrational (infrared and Raman) spectroscopies have seen great interest and rapid development in the recent years as cost-efficient and fast at-line and online analytical techniques for obtaining detailed chemical information for bioprocess development, requiring only a minimal amount of sample [9–11]. They provide comprehensive multivariate chemical information due to simultaneous measurement of the molecular constituents involved in the bioprocess via detection of their specific molecular vibrations. They generate multi-analyte data on various chemical constituents of substrates, intra- and extracellular metabolites, and the overall chemical composition of microbial biomass. We have already demonstrated the efficiency of vibrational spectroscopy in a number of fermentation studies involving, bacteria, yeasts, thraustochytrids and other microorganisms [12, 13]. For example, we have demonstrated that Fourier transform infrared (FTIR) and FT-Raman measurements of bulk samples can obtain all major biochemical constituents of microbial biomass such as lipids, proteins, polyphosphates, and cell wall polysaccharides [14–21]. Moreover, we have demonstrated that FTIR and optical photothermal infrared (O-PTIR) microspectroscopies can provide comprehensive spatial and chemical information on lipogenesis in oleaginous fungi, particularly with regard to lipid content and lipid chemistry [22–24].

As vibrational spectroscopies require only minimal sample amounts, they are particularly suitable for studies involving expensive growth media based on stable isotopes. The combination of vibrational spectroscopies with stable isotope labelling is a powerful methodology for detecting, differentiating and monitoring metabolic pathways [25, 26]. Since the molecular vibration frequency depends on weight of the involved nuclei, molecules with different isotopes of a same element will have different vibrational frequencies. For example, when C^12^ isotopes are substituted by C^13^ isotopes in a typical organic molecule, the band positions of the corresponding molecular vibrations will usually shift to the red (i.e. shift to lower wavenumbers) by 10–40 wavenumbers [27, 28]. This approach has been used to study lipid production in yeasts [27, 28], characterization of single bacterial cells and bacterial communities [26, 29–37], growth rates measurements of bacteria [38, 39], glucose accumulation and protein dynamics in filamentous fungi [40], and characterization of mammalian tissue [41]. Although vibrational spectroscopies enable the simple tracing of the metabolic fate of substrates labelled with different stable isotopes within microorganisms, this technique has never been used to study carbon utilisation in fermentations with mixed carbon substrates. The likely reason is the difficulty of interpreting vibrational spectra in mixed-substrate systems (where signals from many metabolites overlap with isotopic shifts) combined with the high cost and technical complexity of such experiments. Consequently, most studies have focused on simpler single-substrate setups, and therefore workflows for mixed-substrate fungal fermentations have not yet been established.

Thus, for the first time, we assessed stable isotope labelling with infrared and Raman spectroscopies, in bulk and microscopy settings, for studying carbon utilisation in fermentations with mixed carbon substrates. As a model system, we studied carotenogenic and oleaginous Mucoromycota filamentous fungus *Mucor circinelloides*. *Mucor circinelloides* is able to co-produce several valuable metabolites, including lipids, chitin and chitosan, polyphosphates, and carotenoid pigments [16, 17, 42, 43]. Moreover, it is known that Mucoromycota fungi can assimilate various carbon sources, and the preferences cannot be reliably deduced from their isolation origin, despite some similarities within the fungal families [44]. For example, it is known that *M. circinelloides* has the metabolic machinery to utilize glycerol as a substrate [45]. In addition, the literature is sparse regarding the metabolic flux of mixed carbon sources and their subsequent or simultaneous incorporation into fungal biomass constituents, such as proteins, cell wall polysaccharides, carotenoids, and lipids. Our study sheds light on the metabolic tracking of carbon coming from various carbon substrates. In addition, we provide the full biochemical profile of the biomass and the spatial distribution of metabolites utilising multi modal spectroscopic approach. Considering that these metabolites are produced during different growth phases, identifying the preferred carbon source for both exponential and stationary growth phases, and adjusting the concentration profiles accordingly could enable highly precise fermentation processes. In this study, *M. circinelloides* was grown on various ratios of glucose and glycerol carbon source, and fixed amount of yeast extract as a nitrogen source. Glucose was selected as a readily metabolizable, high-energy carbohydrate that supports rapid fungal growth and biomass accumulation, while glycerol (an abundant and low-cost by-product of biodiesel, fish oil and oleochemical industries) was chosen for its potential to enhance lipid accumulation and reduce cultivation costs. Using these two carbon sources in various combinations allowed us to investigate their individual and synergistic effects on growth kinetics and metabolic profiles of *M. circinelloides*, with relevance for sustainable bioprocess development. A nitrogen limited condition was selected in order to trigger the lipid accumulation in the stationary growth phase. Fungal biomass was harvested at six different time points and analysed using a combination of FTIR and FT-Raman methods to measure bulk samples (macro-sampling), as well as FTIR and Raman microspectroscopies to obtain imaging data. This multi-modal approach allowed for both comprehensive bulk characterization and spatially resolved insights into the biochemical composition and distribution within the biomass.

## Materials and methods

### Strain and cultivation

Fungal strain *Mucor circinelloides* VI04473 was provided by the Veterinary institute of the Norwegian University of Life Sciences, Ås, Norway. The selection of the fungal strain is based on our previous studies, which described in detail the carotenogenic and oleaginous properties of large set of strains [15, 16, 21, 46]. The cultivation was performed in two steps: first, agar plate cultivation was performed to obtain fresh spores from the cryopreserved culture; second, submerged cultivation was conducted in liquid media using microtiter plates. *Mucor circinelloides* was cultivated on malt extract agar (MEA, Merck, Germany) for 5 days at 25 °C. Spores were collected from agar plates using 10 ml of sterile saline solution and a bacteriological loop. Submerged cultivation was performed in Duetz-MTPS (Enzyscreen, Heemstede, The Netherlands), consisting of 24‐square well polypropylene microtiter plates (MTPs), with low evaporation sandwich covers with a clamp system.

The growth media contained either isotopically labelled (^13^C) or standard (^12^C) glucose and/or glycerol in the amounts stated in Table 1.

**Table 1:** Concentration of carbon sources in g/L

Sample name	glucose (^12^C)	glucose (^13^C)	glycerol
*nGlu*	40	-	-
*iGlu*	-	40	-
*nGlu1:Gly1*	20	-	20
*iGlu1:Gly1*	-	20	20
*nGlu1:Gly7*	5	-	35
*iGlu1:Gly7*	-	5	35
*Gly*	-	-	40

In addition to the carbon sources, all samples contained the following nutrients (in g/L): yeast extract 3, KH_2_PO_4_ 7, Na_2_HPO_4_ 2, MgSO_4_·7H_2_O 1.5, CaCl_2_·2H_2_O 0.1, FeCl_3_·6H_2_O 0.008, ZnSO_4_·7H_2_O 0.001, CoSO_4_·7H_2_O 0.0001, CuSO_4_·5H_2_O 0.0001, and MnSO_4_·5H_2_O 0.0001.

Following the preparation of the media by mixing the components, 3 ml of sterile media was transferred into the autoclaved microtiter plates, and each well was inoculated with 20 µL of the spore suspension. Cultivations were performed at 28 °C and 405 rpm agitation speed (1.9 cm circular orbit) in a MaxQ 4000 shaker (Thermo Scientific, United States). Following timepoints of cultivation were examined (in h): 8, 14, 24, 36, 60, 120. One well per each timepoint and media was prepared, summing up to 42 samples.

### Preparation of fungal biomass for vibrational spectroscopy analyses

Culture broths were collected from MTPs using a sterile plastic Pasteur pipette. The collected culture broth was split in two parts: (1) 1 mL was centrifuged (5 min, 8000 rpm) to separate the culture medium and biomass. Subsequently the biomass was washed 3 times using distilled water. The biomass was freeze-dried for 24 h in order to estimate the biomass concentration. (2) The remaining 2 mL of culture broth were gently washed with distilled water on filter (0.22 μm, Milipore) using a vacuum pump in order prevent any damage to hyphae. Approx 1–2 mg of freshly washed biomass was resuspended in distilled water and deposited onto microscope slides directly prior the microspectroscopy analyses. The remaining fresh washed biomass was stored in Eppendorf tubes in the freezer at -20 °C until the FTIR and FT-Raman bulk biomass measurements.

### Microscopy

Micrographs were obtained from fresh biomass in bright-field mode with a DM6B microscope (Leica Microsystems, Wetzlar, Germany) with 40× magnification.

### Raman spectroscopies

The FT-Raman spectra of bulk biomass were recorded in backscattering geometry using a MultiRAM FT-Raman spectrometer (Bruker Optik GmbH, Germany) equipped with a neodymium-doped yttrium aluminium garnet (Nd: YAG) laser (1064 nm, 9394 cm-1), and germanium detector cooled with liquid nitrogen. For each measurement, 0.5–1 mg of freeze-dried sample was deposited in aluminium sample container and pressed with pestle. The spectra were recorded with a total of 128 scans, using Blackman–Harris 4-term apodization, spectral resolution of 4 cm^− 1^, with a digital resolution of 1.928 cm^− 1^, over the range of 3785–50 cm^− 1^, at 500 mW laser power. In some cases (in particular, for early cultivation times and for some samples grown in glycerol media), to prevent heating and burning effects, the measurements were conducted by using reduced laser power (150–300 mW). Each biomass sample was analysed in three technical replicates, resulting in 126 spectra in total. The OPUS 8.1 software (Bruker Optik GmbH, Germany) was used for data acquisition and instrument control.

The Raman microspectroscopy spectra were recorded in backscatter mode using a alpha300R confocal Raman microscope (WITec GmbH, Germany) equipped with 785 nm excitation laser with 125 mW output power, Ultra-High-Throughput UHTS400 spectrometer with deep depletion charge-coupled device (CCD) detector and 300 & 1200 grooves/mm gratings, and with a 100× objective with 0.9 numerical aperture and 1.00 mm working distance (Carl Zeiss AG, Germany). Fresh fungal biomass was suspended in distilled water, 10 µl of the suspension was pipetted onto a 1 mm thick IR transparent calcium fluoride (CaF_2_) microscope slide (window) and dried at room temperature for 1 h. Each individual Raman microspectroscopy spectrum (point measurement) was recorded with a grating of 300 grooves/mm, within spectral range of 2920–0 cm^− 1^, and 20 accumulations with 3s integration time each. Each biomass sample was measured at least 6 times (maximum 53 times), resulting in 682 spectra in total. The spectral maps were acquired as spectral image, with a grating of 300 grooves/mm, within the spectral range of 2920–0 cm^− 1^, with 0.5–1.0 s integration time, and with a step size of 0.25 μm in x and y directions. Visible images of the measured samples were obtained by a CCD camera coupled to the microscope. The Control Five 5.3 software (WITec GmbH, Germany) was used for data acquisition and instrument control.

### FTIR spectroscopies

The FTIR transmittance spectra of bulk fungal biomass were measured using a High Throughput Screening eXTension (HTS-XT) unit coupled to a Vertex 70 FTIR spectrometer (both Bruker Optik, Germany). The spectroscopy system was equipped with a globar mid-IR source and a deuterated L-alanine doped triglycene sulphate (DLaTGS) detector. For each measurement, 10 µl of homogenized fungal biomass was pipetted onto an IR transparent 384-well silicon microplate and dried at room temperature for 1 h. The HTS-FTIR spectra were recorded with a total of 64 scans, using Blackman–Harris 3-term apodization, spectral resolution of 6 cm^− 1^, and digital spacing of 1.928 cm^− 1^, over the range of 4000–400 cm^− 1^, and an aperture of 6 mm. Spectra were recorded as the ratio of the sample spectrum to the spectrum of the empty IR transparent microplate. Each biomass sample was analysed in three technical replicates, resulting in 126 spectra in total. The OPUS 8.2 software (Bruker Optik GmbH, Germany) was used for data acquisition and instrument control.

The FTIR microspectroscopy transmittance spectra were measured using a Hyperion 3000 infrared microscope coupled to a Vertex 70 FTIR spectrometer (both Bruker Optik, Germany). The spectroscopy system was equipped with a 15× Schwarzschild objective with 0.4 numerical aperture and 24.00 mm working distance, a globar mid-IR source, and a deuterated L-alanine doped triglycene sulphate (DLaTGS) detector. Fresh fungal biomass was suspended in distilled water, 10 µl of the suspension was pipetted onto a 1 mm thick IR transparent zinc selenide (ZnSe) microscope slide (window) and dried at room temperature for 1 h. The µFTIR spectra were recorded with 128 × 128 mercury cadmium telluride (MCT) focal plane array (FPA) liquid nitrogen-cooled detector, and were recorded with a total of 256 scans, using Blackman–Harris 3-term apodization, spectral resolution of 8 cm^− 1^, and digital spacing of 3.851 cm^− 1^, over the range of 3850–900 cm^− 1^, and a fully open aperture. Spectra were recorded as the ratio of the sample setup to the sample-free setup of the empty IR transparent microscope slide. Visible images of the measured samples were obtained by a CCD camera coupled to the microscope. Each biomass sample was measured as one hyperspectral image containing 16,384 spectra, resulting in 42 hyperspectral images in total. The OPUS 8.2 software (Bruker Optik GmbH, Germany) was used for data acquisition and instrument control.

### Spectral preprocessing

The spectral data sets were preprocessed to remove baseline variations and intensity differences, and to enhance the chemical signals and supress interferents [47]. These unwanted variations arise due to non-ideal instrument and sample properties, such as fluorescence- and overheating-related baseline offsets in FT-Raman and differences in the effective optical path length between sample dry films in FTIR (due to slight differences in sample concentration). All preprocessing methods and data analyses were performed using Aspen Unscrambler 14.2 (Aspen Technology, Inc, USA), Control Five 5.3 software (WITec GmbH, Germany), and Orange data mining toolbox version 3.36 (University of Ljubljana, Slovenia) [48, 49].

The HTS-FTIR dataset was preprocessed with two different procedures: (1) For estimates of the accumulation of lipids and polyphosphates based on the intensities of IR bands at 1746 and 1700 cm^− 1^ (related to C = O stretching vibration for ^12^C and ^13^C, respectively) and at 1263 cm^− 1^ (related to P = O stretching vibration), the following sequential procedure was used: (i) truncation of data to the 1800 –800 cm^− 1^ region, (ii) linear baseline correction, (iii) band area normalization (1670 –1600 cm^− 1^, amide I band C = O stretching vibration in amides, which is predominantly associated with proteins), (iv) conversion into second derivatives by using SG algorithm (polynomial 2, window size 15, derivative order 2). (2) For principal component analysis (PCA) and consensus PCA (CPCA), the following sequential procedure was used: (i) conversion into second derivatives by using SG algorithm (polynomial 2, window size 11, derivative order 2), (ii) truncation of data to 3200 − 2600 and 2000 –600 cm^− 1^ regions, (iii) normalization by multiplicative signal correction (MSC).

The FT-Raman dataset was preprocessed with three different procedures: (1) For estimates of the accumulation of lipids and carotenoids based on the intensities of Raman bands at 2855 cm^− 1^ (related to C-H stretching vibration in -CH_2_) and at 1525 cm^− 1^ (related to -C = C- stretching), the following sequential procedure was used: (i) conversion into second derivatives by using SG algorithm (polynomial 2, window size 15, derivative order 2), (ii) truncation of data to 3200 − 2500 and 1800 –600 cm^− 1^ regions, (iii) normalization by multiplicative signal correction (MSC). (2) For PCA and CPCA, the following sequential procedure was used: (i) rubberband correction, (ii) conversion into second derivatives by using SG algorithm (polynomial 2, window size 11, derivative order 2), (iii) truncation of data to 1800 –400 cm^− 1^ region, (iv) vector normalization. (3) For estimating total lipid and total phosphorus contents based on PLSR models, the following sequential procedure was used: (i) smoothing by using SG algorithm (polynomial 2, window size 15, derivative order 0), (ii) rubber band baseline correction, (iii) truncation of data to 3200 − 2400 and 1900 –500 cm^− 1^ regions, (iv) normalization by extended multiplicative signal correction (EMSC), an MSC model extended by a linear, quadratic, and cubic components.

The FTIR microspectroscopy dataset was preprocessed with two different procedures: (1) For creating hyperspectral images, the pixel-spectra were scatter corrected using a deep convolutional neural network (DCNN), a deep learning-based scattering correction model, trained on data simulated using electromagnetic scatter theory exact [50–52]. Establishing the scatter correction model was done as described by Muthreich et al. [52], but with numerical aperture in range 0.20–0.45, corresponding to the one set by the pixel size in the microscpectroscopic image. (2) For PCA, the following sequential procedure was used: (i) removal of noisy pixels (spectra) from empty or nearly empty regions as described by Tafintseva et al. [53], (ii) binning of the remaining pixel-data into blocks covering 10 × 10 pixel (27 × 27 µm^2^) regions (for the image edges the following size blocks were used 10 × 8, 8 × 10 and 8 × 8 pixels) and removal of blocks with less than 5 pixels, (iii) conversion into second derivatives by using SG algorithm (polynomial 2, window size 9, derivative order 2), (iv) normalization by extended multiplicative signal correction (EMSC), an MSC model extended by a linear and quadratic components [54, 55] where the refence spectrum was mean of all block-spectra.

The point measurement Raman microspectroscopy dataset was preprocessed by the following sequential procedure: (i) cosmic ray removal, (ii) rubber band baseline correction, (iii) truncation of data to 1800 –400 cm^− 1^ region, (iv) normalization by area normalization. The Raman microspectroscopy imaging dataset was preprocessed by the following sequential procedure: (i) cosmic ray removal, (ii) polynomial baseline correction, (iii) truncation of data to 1800 –400 cm^− 1^ region.

### Principal component analysis and basis analysis

Biochemical similarities between samples were estimated by using principal component analysis (PCA) and consensus PCA (CPCA). CPCA was used on multiblock spectral data, consisting of preprocessed HTS-FTIR and FT-Raman data blocks. In the CPCA, technical replicates were averaged after the preprocessing in order to obtain sample-to-sample correspondence between the data blocks [56, 57]. For the PCA of the FTIR microspectroscopy dataset, the PCA space was established by decomposing the preprocessed binned spectra from all 42 hyperspectral images. The PCA scores plots for the FTIR microspectroscopy dataset were plotted on this common PCA space.

Estimates of total lipid and total phosphorus contents for two growth conditions were based on our previously established PLSR models [15] for nonderivative FT-Raman spectral data. They were calculated only for samples belonging to growth conditions *nGlu* and *nGlu1:Gly1* (excluding 8 h timepoints), because for those samples prediction residuals had low values. Prediction residuals are the amount of spectral variation not explained by the PLSR model when projecting the sample. Large residuals indicate the sample lies outside the calibration model and its prediction may be unreliable.

The Raman microspectroscopy images were obtained following the basis analysis according to Schmidt et al. [58], where each of the recorded spectra from the imaging dataset was fitted as a linear combination of *basis spectra* using least squares method with a basis analysis algorithm (*True component analysis* in Control Five 5.3 software). The analyses with up to four components were conducted. Bands related to proteins, carbohydrates, lipids, polyphosphates and carotenoids were assigned in the basis spectra. Following the fitting procedure, images for components were constructed by using the fitting factors.

## Results and discussion

### Biomass production

The morphology of fungal biomass exhibited thin hyphae at the beginning of the cultivations of all samples. Gradually, the hyphal branching occurred during an 8–14 h period, accompanied by the thickening of the cell wall and hyphae. Lipid body formation was detected as early as at 14 h. In addition, yeast-like cells started to appear in all samples between 24 and 36 h. By the end of the cultivation period, all samples contained substantial amounts of lipid bodies, except for those grown under glycerol conditions. Generally, hyphae of glycerol-grown samples were notably thinner, and this biomass became aggregated by 60 h, showing signs of sporangium formation, likely due to unfavourable growth conditions.

**Fig. 1:**
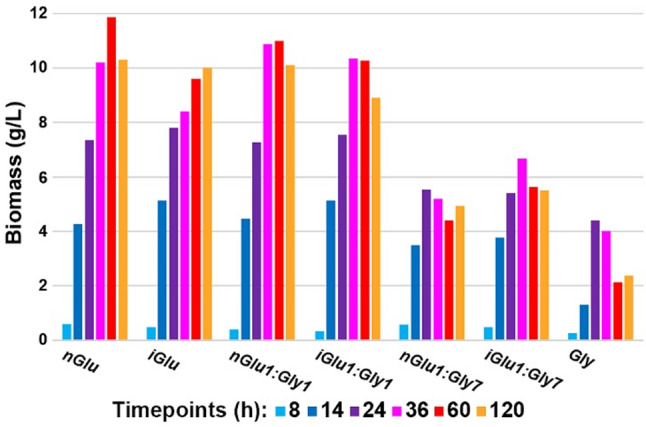
Biomass production during fermentations. Colors represent different sampling timepoints: light blue 8 h, dark blue 14 h, violet 24 h, magenta 36 h, red 60 h and orange 120 h. Samples are grouped according to the carbon source in the growth medium: *nGlu* glucose (^12^C) 40 g/L; *iGlu* glucose (^13^C) 40 g/L; *nGlu1:Gly1* glucose (^12^C) 20 g/L and glycerol 20 g/L; *iGlu1:Gly1* glucose (^13^C) 20 g/L and glycerol 20 g/L; *nGlu1:Gly7* glucose (^12^C) 5 g/L and glycerol 35 g/L; *iGlu1:Gly7* glucose (^13^C) 5 g/L and glycerol 35 g/L; *Gly* glycerol 40 g/L

We used growth media with a concentration of 40 g/L of either glucose or glycerol, as well as a mixed carbon source containing a 1:1 or 7:1 weight ratio of glucose and glycerol. Both ^12^C glucose and glycerol contain approximately 40%_w/w_ carbon, with 43%_w/w_ in ^13^C glucose, thus all media had similar carbon content. Yeast extract was included as the sole source of nitrogen and it was anticipated to be used for the initial biomass production due to its high nitrogen and micro- and macronutrient content. Yeast extract aimed to support early growth by providing amino acids and other essential nutrients, facilitating the rapid onset of fungal cells proliferation observed in the initial stages of fermentation. Although yeast extract could serve as an additional carbon source since it contains approx. 40% of carbon, its contribution to the final biomass constituents was relatively minor compared to the primary carbon sources of glucose and glycerol, due to its low content in the growth media and its rapid consumption. Furthermore, the carbon in yeast extract is predominantly bound in amino acids and small peptides and is therefore not readily diverted into the production of lipids.

The limited availability of the ¹³C-labeled glucose substrate prevented us from conducting the experiment with bioreplicates, and we acknowledge that this could be considered a shortcoming. However, our results showed very similar outcomes between isotopically labelled (^13^C) and standard (^12^C) media (Fig. 1). Moreover, these results are very well aligned with our previous studies, where the mean of biomass concentration of 11 bioreplicates of *Mucor circinelloides* grown in the principally same growth media (with 80 g/L glucose content, though this surplus was not consumed) was 10.08 g/L (range: 9.35–11.52 g/L, median: 9.90 g/L), with 0.62 g/L standard deviation, and 0.19 g/L standard error [18]. The biomass production in the mixed carbon source media with equal amount of glucose and glycerol (*nGlu1:Gly1* and *iGlu1:Gly1*) was very similar to the production in pure glucose media (*nGlu* and *iGlu*), with final biomass concentrations up to approximately 10 g/L. A significant drop in biomass production was observed for pure glycerol media (*Gly*), with concentrations up to approximately 4 g/L, compared to the pure glucose media, and significant reduction in the biomass concentration after 36 h. Notably, the small addition of glucose to glycerol media (*nGlu1:Gly7* and *iGlu1:Gly7*) resulted in approx. 50% increase in biomass production, compared to the pure glycerol media. We wish to emphasize that, although biological replicates were not used in this study, all spectra were recorded in at least three technical replicates for each technique (three replicates each for FTIR and FT-Raman bulk measurements, and even more technical replicates for Raman and FTIR microspectroscopy), ensuring that the results are based on statistically sufficient replicate measurements.

### Vibrational spectra of bulk biomass of Mucor circinelloides

**Fig. 2:**
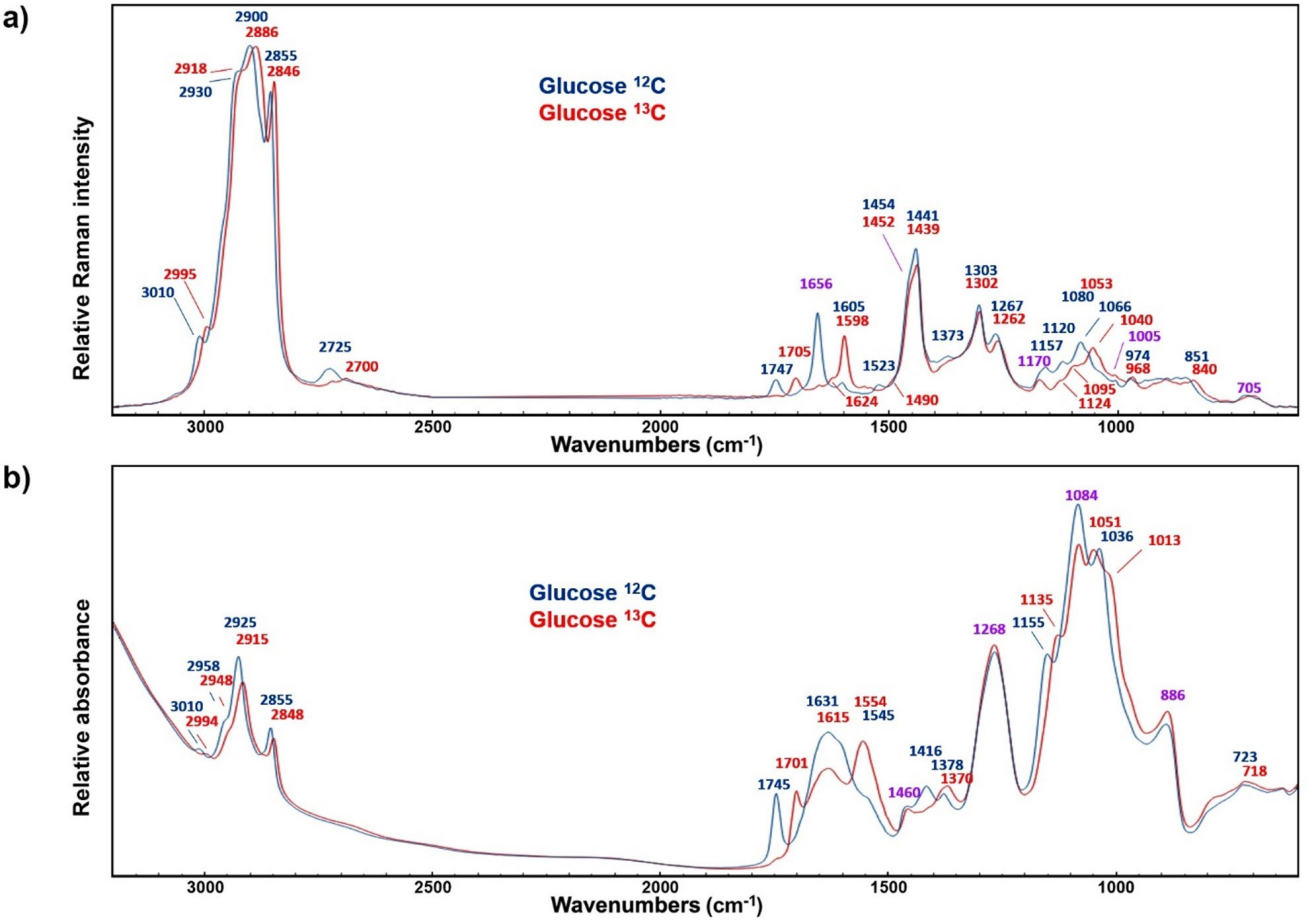
Vibrational spectra of bulk biomass of *Mucor circinelloides.* **a** FT-Raman and **b** FTIR spectra of fungal biomass grown for 60 h in glucose control media containing either isotopically labelled (^13^C; blue) or standard (^12^C; red) glucose. Spectra were normalised by vector normalization (Raman) and EMSC (FTIR)

Both FTIR and FT-Raman spectroscopies are effective techniques for monitoring and differentiating metabolites based on stable isotope substrates [32]. The representative FT-Raman and FTIR spectra of the fungal biomass grown in control media containing either isotopically labelled (^13^C) or standard (^12^C) glucose at the midpoint (60 h) of the fermentation show characteristic signals of principal metabolites (Fig. 2). It is evident that most of the signals of biomass grown in the isotopically labelled condition exhibit a red shift (i.e. a shift to lower wavenumbers) due to the incorporation of the heavier ^13^C isotope into the metabolites instead of the lighter standard ^12^C. Table 2 provides a detailed overview of the Raman and infrared bands corresponding to fungal metabolites.

The most intensive Raman bands are associated with triglyceride (TAG) lipids: =C-H stretching, C-H stretching in -CH_3_ and -CH_2_, C=O stretching in esters, C=C stretching, C-CH_2_, and CH_3_ deformations, C-C and C-O stretching and C=C deformations. Protein-related bands are relatively weak, and, in case of ^12^C glucose media, they overlap with lipid bands: amide I C = O stretching, C=C phenyl ring vibration, amide III N-H bending and C-N stretching, phenyl ring deformation. Protein-rich biomass is synthesized during the initial phase of the fermentation process (the first 8 h), primarily through the consumption of amino acids in yeast extract. Since the yeast extract contains the standard ^12^C isotope, weak ^12^C-based protein signals are also present in the biomass grown in media containing ^13^C glucose, with the most prominent signal appearing at 1660 cm^− 1^. Most of the Raman bands related to cell wall carbohydrates, namely glucosamines, glucans, and glucuronans, are common with the bands of lipids and proteins, including C-H stretching vibrations, C=O stretching in esters and amides, and CH_2_ and CH_3_ deformations. However, during the lipid-accumulation phase of microbial growth, these bands are predominantly associated with lipids rather than carbohydrates. In addition to these bands, carbohydrates have specific bands, such as C-C, C-O, C-O-C, C-N, C-H, COH stretching, deformations, and combination bands. Carotenoid bands in Raman spectra are significantly enhanced by the resonant Raman effect when the excitation wavelength is close to the electronic absorption band of carotenoids, resulting in increased signal intensity and improved detection sensitivity. Although carotenoid bands are more prominent when measurements were conducted with a 785 nm excitation laser of the Raman microspectrophotometer (see below) than with a 1064 nm excitation laser of the FT-Raman spectrophotometer, they were nevertheless observable in the FT-Raman spectra: -C = C- stretching, and -C-C- stretching and CH deformation. The spectral bands associated with polyphosphates, namely P = O stretching and P-O-P stretching, are weak and overlapping with bands of other metabolites. However, in the spectra of biomass grown in media containing ^13^C glucose, the P = O stretching at 1170 cm^− 1^ is clearly visible due to the red shift of the carotenoid-related band at 1124 cm^− 1^. The assignment of the Raman bands was done according to our previously published study and the references therein [15].

Similarly as in the Raman spectra, triglyceride lipids have very prominent IR bands associated with: =C-H stretching vibrations, C-H stretching in -CH_3_ and -CH_2_, C=O stretching in esters, -CH_2_, and -CH_3_ deformations, C-O-C stretching, and -CH_2_ deformations. The strongest protein-related IR bands are: amide I C=O stretching vibrations, and amide II N-H bending and C-N stretching vibration. As mentioned previously, protein-rich biomass is primarily synthesized through the consumption of yeast extract during the initial phase of the fermentation process, and since the yeast extract contains the standard ^12^C isotope, ^12^C-based protein signals are also present in the biomass grown in media containing ^13^C glucose. The most prominent IR bands related to cell wall carbohydrates, such as glucans, chitin and chitosan, overlap with the bands of lipids and proteins, including C-H stretching vibrations, C=O stretching in esters and amides, and CH_2_ and CH_3_ deformations. As mentioned earlier for the complementary Raman bands, during the lipid-accumulation phase of microbial growth, these IR bands are predominantly associated with lipids rather than carbohydrates. In addition to these bands, carbohydrates have specific bands, such as C-O-C stretching, and C-O-C and COH deformations. The spectral bands associated with polyphosphates have much stronger signals in the IR spectra than in the FT-Raman spectra. Specifically, P = O stretching, P-O stretching and P-O-P stretching are clearly visible. The assignment of the IR bands was done according to our previously published study and the references therein [17].

**Table 2:** Raman and infrared band assignments for fungal biomass constituents

	Raman band (cm^− 1^)			Infrared band (cm^− 1^)		
	Molecular vibration	^12^C	^13^C	Molecular vibration	^12^C	^13^C
Triglyceride lipids (TAGs)	=C-H stretching	3010	2995	=C-H stretching	3010	2994
	C-H stretching in -CH_3_ and -CH_2_	2930	2918	C-H stretching in -CH_3_ and -CH_2_	2958	2948
		2900	2886		2925	2915
		2855	2846		2855	2848
		2725	2700			
	C = O stretching in esters	1747	1705	C = O stretching in esters	1745	1701
	C = C stretching	1656	1598			
	C-CH_2_, and CH_3_ deformations	1454	1452	C-CH_2_, and CH_3_ deformations	1460	1460
		1442	1439		1416	1382
		1373	1370		1378	1370
		1303	1302			
	C-C and C-O stretching	1081	1055	C-O-C stretching	1155	1135
		1066	1040	-CH_2_ deformations	723	718
	C = C deformations	973	968			
Proteins	amide I C = O stretching	1660	1624	amide I C = O stretching	1640	1615
	C = C phenyl ring	1605	1565	amide II N-H bending and C-N stretching vibration	1550	1540
	amide III N-H bending and C-N stretching	1268	1262			
	phenyl ring deformation	1005	966			
Cell wall carbohydrates (glucosamines, glucans, and glucuronans)	C-H stretching in -CH_3_ and -CH_2_	2930	2918	C-H stretching in -CH_3_ and -CH_2_	2958	2948
		2900	2886		2925	2915
		2855	2846		2855	2848
		2750	2700			
	C = O stretching in esters (glucuronans)	1745	1705	C = O stretching in esters (glucuronans)	1745	1705
	amide I C = O stretching (chitin)	1650	1625	amide I C = O stretching (chitin)	1650	1615
	-CH_2_ and CH_3_ deformations	1442	1439	-CH_2_ and CH_3_ deformations	1460	1460
		1373	1370		1416	1382
		1303	1302		1378	1370
	C-C, C-O, C-O-C, C-N, C-H, COH stretching, deformations, and combination bands	1127	1095	C-O-C stretching and C-O-C and COH deformations	1155	1135
		940	906		1080	1051
		851	840		1036	1013
Polyphosphates	P = O stretching	1170		P = O stretching	1268	
				P-O stretching	1084	
	P-O-P stretching	700		P-O-P stretching	886	
Carotenoids	-C = C- stretching	1523	1490	Not detectable (low concentration in fungal biomass)		
	-C-C- stretching and CH deformation	1157	1124			

**Fig. 3:**
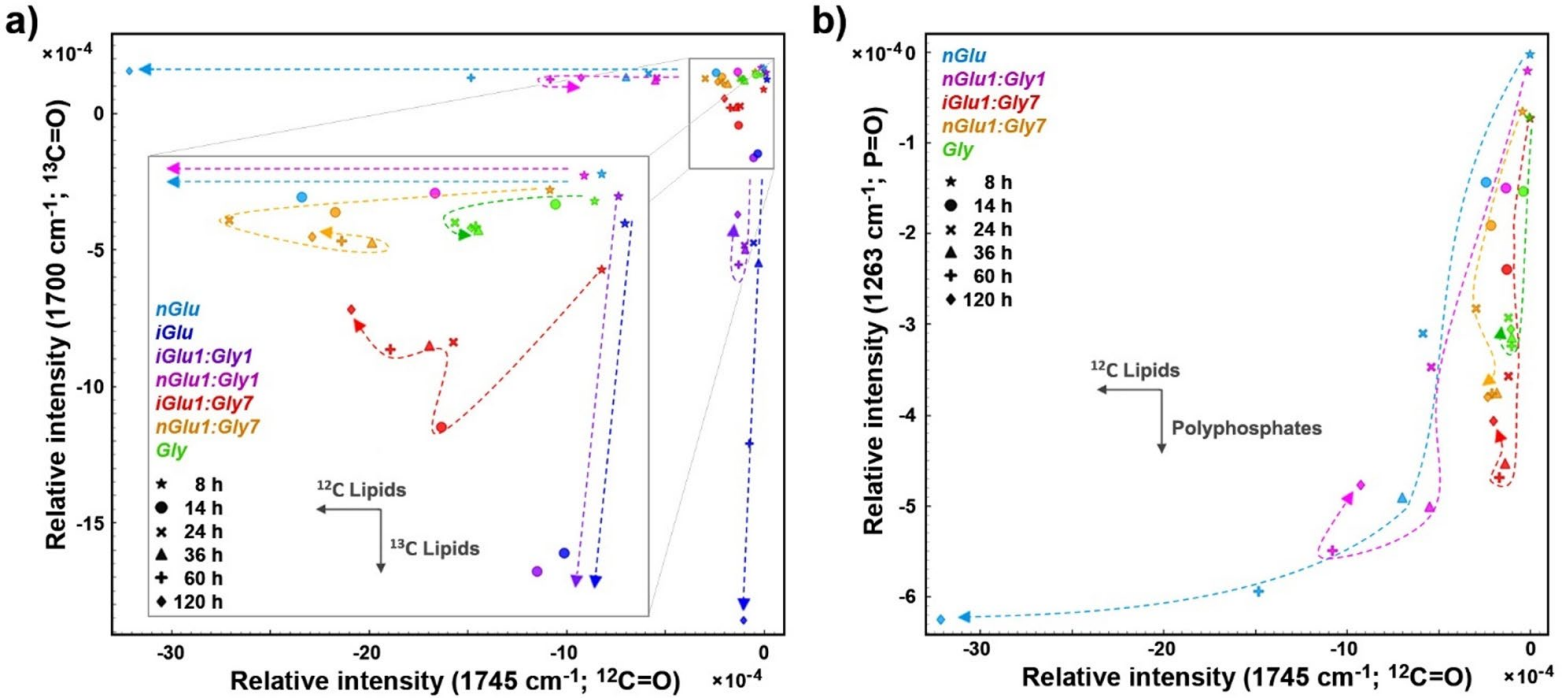
Estimates of the accumulation of lipids and polyphosphates based on the intensities of IR bands related to: **a** C=O (for ^12^C and ^13^C) and **b** P=O stretching vibrations. High negative intensity values correspond to the high absorbance as the spectra were converted into second derivatives. The vectors are approximating the increase in the relative amount of metabolites: lipids and polyphosphates; ^12^C and ^13^C indicates carbon isotope composition. The dashed lines represent approximations of temporal progression. The spectra were averaged, and the data of the samples corresponding to *iGlu* and *iGlu1:Gly1* media are omitted from (**b**) for clarity

Specific bands in FTIR and FT-Raman spectra can be used as valuable indicators for estimating the presence and concentration of major metabolites within a sample. Here, lipid and polyphosphate accumulation were quantified by normalizing the FTIR spectra to the amide I band, which is predominantly associated with protein content, and evaluating the intensities of the C=O stretching band, indicative of TAG lipid content, and the P = O stretching band, representative of polyphosphate content (Fig. 3). The fungal biomasses grown in control media containing either isotopically labelled (^13^C) or standard (^12^C) glucose were accumulating lipids and polyphosphates until the final point of the fermentation (120 h). The biomasses grown in mixed media with equal amount of glucose and glycerol (*nGlu1:Gly1* and *iGlu1:Gly1*) were accumulating lipids and polyphosphates until approx. the midpoint of the fermentation (60 h), after which these energy reserves were gradually depleted without apparent utilisation of glycerol. However, the biomasses grown in mixed media with a large surplus of glycerol (*nGlu1:Gly7* and *iGlu1:Gly7*) show significant accumulation of polyphosphates until approx. 60 h of fermentation, as well as signs of modest utilisation of glycerol for lipid production after approx. 36 h of fermentation. The biomass composition of fungi grown in mixed media with isotopically labelled (^13^C) glucose (*iGlu1:Gly7*) shows that lipid accumulation during the first 14 h is based on glucose (as indicated by ^13^C = O stretching signals), followed by lipid depletion once all the glucose (^13^C) in the media is consumed, and finally lipid accumulation based on glycerol after approx. 36 h (as indicated by ^12^C = O stretching signals). This realisation can be considered as highlight of the proposed methodology, as we precisely identified the flux of mixed carbon sources to exact metabolite. The biomass grown in pure glycerol shows very modest accumulation of lipids and somewhat stronger accumulation of polyphosphates until approx. 24 h of fermentation, followed by a decrease of both metabolites. All samples demonstrated substantial accumulation of polyphosphates, particularly when glucose was not available (*Gly*) or is available in limited supply (*nGlu1:Gly7* and *iGlu1:Gly7*), suggesting that polyphosphates serve as significant energy reserves under glucose-limited conditions.

It is known that filamentous fungi exhibit different preferences to various substrates, and that they can alter their metabolism to ensure efficient nutrient uptake. This adaptability originates from the fact that the natural environment of filamentous fungi is nutritionally complex. Consequently, energetically less demanding substrates are typically preferred [59]. In our study, when glucose was limited, the fungus experiences a reduced influx of readily usable carbon for ATP generation via glycolysis. In response, cells often accumulate polyphosphates as alternative energy and phosphate storage molecules. These linear polymers of inorganic phosphate can be mobilized when energy demand increases or nutrient conditions become unfavourable. Polyphosphates are chains of phosphate residues connected by high-energy phosphoanhydride bonds. In fungal cells, polyphosphates can be localized in intracellular vacuoles or granules, as well as in the cell wall [60]. It has been reported that cell wall polyphosphates serve as counter anions to positively charged chitin and chitosan. In addition, the high-energy phosphoanhydride bond act as a source of energy for various metabolic processes and gene functions. Polyphosphates also play a protective role by participating in oxidative stress response pathways, heavy metal chelation, and buffering mechanisms [61]. Therefore, even in the presence of glycerol, when the preferred substrate (glucose) was limited and yeast extract depleted, it is hypothesized that *Mucor circinelloides* experiences nutrient stress and ultimately relies on polyphosphate reserves as the major intracellular energy source. This suggests that glycerol alone was insufficient to meet the energy demands under these conditions, likely due to limited metabolic activation or transport inefficiencies. Previously it has been reported that *M. circinelloides* possesses inducible glycerol dehydrogenase enzymes, but their expression is repressed in the presence of preferred substrates like glucose [45]. As a result, in mixed carbon cultures, glycerol metabolism may remain inactive unless glucose levels fall below a threshold. This regulatory behaviour likely underlies the limited glycerol utilization observed in our experiments when the glucose level was decreased from being equal to glycerol (Glu1:Gly1) to a large surplus of glycerol (Glu1:Gly7). Future studies should investigate the interaction between phosphorus-based and carbon-based energy storage mechanisms in filamentous fungi grown on mixed carbon sources.

**Fig. 4:**
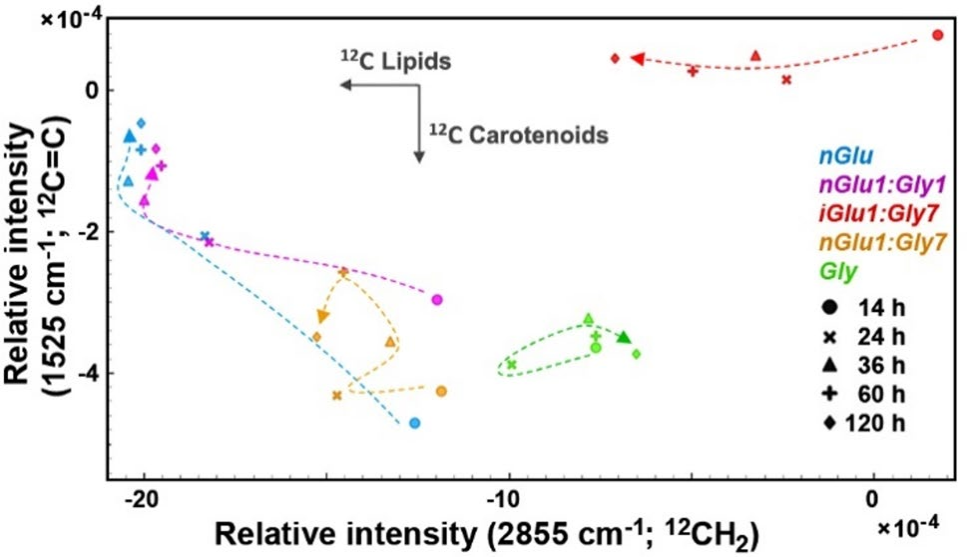
Estimates of the accumulation of lipids and carotenoids based on the intensities of Raman bands related to: C-H stretching in -CH_2_ (at 2855 cm^− 1^ for ^12^C) and -C=C- stretching (at 1525 cm^− 1^ for ^12^C). High negative intensity values correspond to the high Raman intensity as the spectra were converted into second derivatives. The vectors are approximating the increase in the relative amount of metabolites: lipids and carotenoids; ^12^C indicates carbon isotope composition. The dashed lines represent approximations of temporal progression. The spectra were averaged, and the data of the samples corresponding to *iGlu* and *iGlu1:Gly1* media are omitted for clarity since they do not produce metabolites with those bands

As opposed to FTIR spectra, FT-Raman spectra lack a strong protein-specific band that could be used for spectral normalization. Therefore, the spectra were normalized to total biomass (by using MSC). To evaluate the content of TAG lipids and carotenoid pigments, the intensities of the C-H stretching in -CH_2_, indicative of TAG lipid content, and the and -C = C- stretching band, representative of carotenoid content, were used (Fig. 4). The Raman results corroborate the FTIR results, showing that fungi grown in mixed media with a large surplus of glycerol (*nGlu1:Gly7*) begin to deplete the initially accumulated lipids after approx. 24 h, followed by re-accumulation after approx. 36 h. The production of carotenoid pigments is relatively rapid in the early stages of fermentation (the first 14 h), and their content in the biomass decreases as the lipid content increases. The absence of the carotenoid-related band at 1525 cm^− 1^ in the spectra of fungi grown in mixed media with isotopically labelled (^13^C) glucose (*iGlu1:Gly7*) strongly indicates that the carotenoids are primarily derived from glucose rather than yeast extract or glycerol. Our previous study on oleaginous and carotenogenic yeast *Rhodotorula toruloides* has shown similar production dynamics to those observed here, with rapid production of carotenoids during the first 24 h of the fermentation, followed by rapid production of lipids during the subsequent period (48–72 h) of fermentation [13]. It is known that carotenoids and lipids compete for the common metabolic precursor Acetyl-CoA. Carotenoids are produced in the mevalonate pathway, and for lipids, Acetyl-CoA is utilised in fatty acid synthesis (FAS). Thus, inhibition of FAS can result in increased carotenoid production in *Mucor circinelloides.* It can be hypothesised, that once nitrogen starts becoming limited, the triggered lipid accumulation redirects the metabolic flux of acetyl-CoA from mevalonate pathway to FAS. In addition, glycerol has been reported as less favourable carbon source for carotenoid production by *Mucor circinelloides*, compared to glucose [62].

Our published studies demonstrate a very good correlation between IR and Raman spectral variance and biological variance since the spectra accurately represent the biochemical profile of microbial biomass [15–17]. Moreover, IR and Raman spectroscopy measurements of macroscopic samples can be calibrated against reference analyses, such as lipid or carotenoid contents acquired by chromatographies, and serve as a fast, low-cost, quantitative analytical methods [15]. Based on our previously established PLSR models [15], we estimated total lipid and total phosphorus contents for two growth conditions, namely *nGlu* and *nGlu1:Gly1* (Figure S1 in the Supplementary Information). The samples contained the following range of amounts of the total lipids (expressed as a percentage of a dry weight): nGlu 10–46% and nGlu1:Gly1 7–35%, and the following range of amounts of the total phosphorus (expressed as a percentage of a dry weight): nGlu 1.2–3.3% and nGlu1:Gly1 0.3–3.3%. These media were relatively similar to those used in the model study, except for a lower carbon concentration (the model media contained 80 g/L glucose) and a different nitrogen source (ammonium sulphate instead of yeast extract), which could introduce discrepancies because the biomass chemical composition was not identical to that used to build the models. Nevertheless, prediction residuals for samples grown under these two conditions were small, indicating that the PLSR models provided reliable estimates. The only exception was early-stage biomass (8 h), for which residuals were too high to be considered reliable. Although PLSR models for total carotenoids in *Mucor* were also available, these models are reliable only for strains producing relatively high levels of carotenoids (~ 1000 µg/gdry weight), whereas the strain used here produces relatively low amount of carotenoids (~ 200 µg/g_dry weight_), limiting the model’s applicability.

### Multiblock consensus principal component analysis

**Fig. 5:**
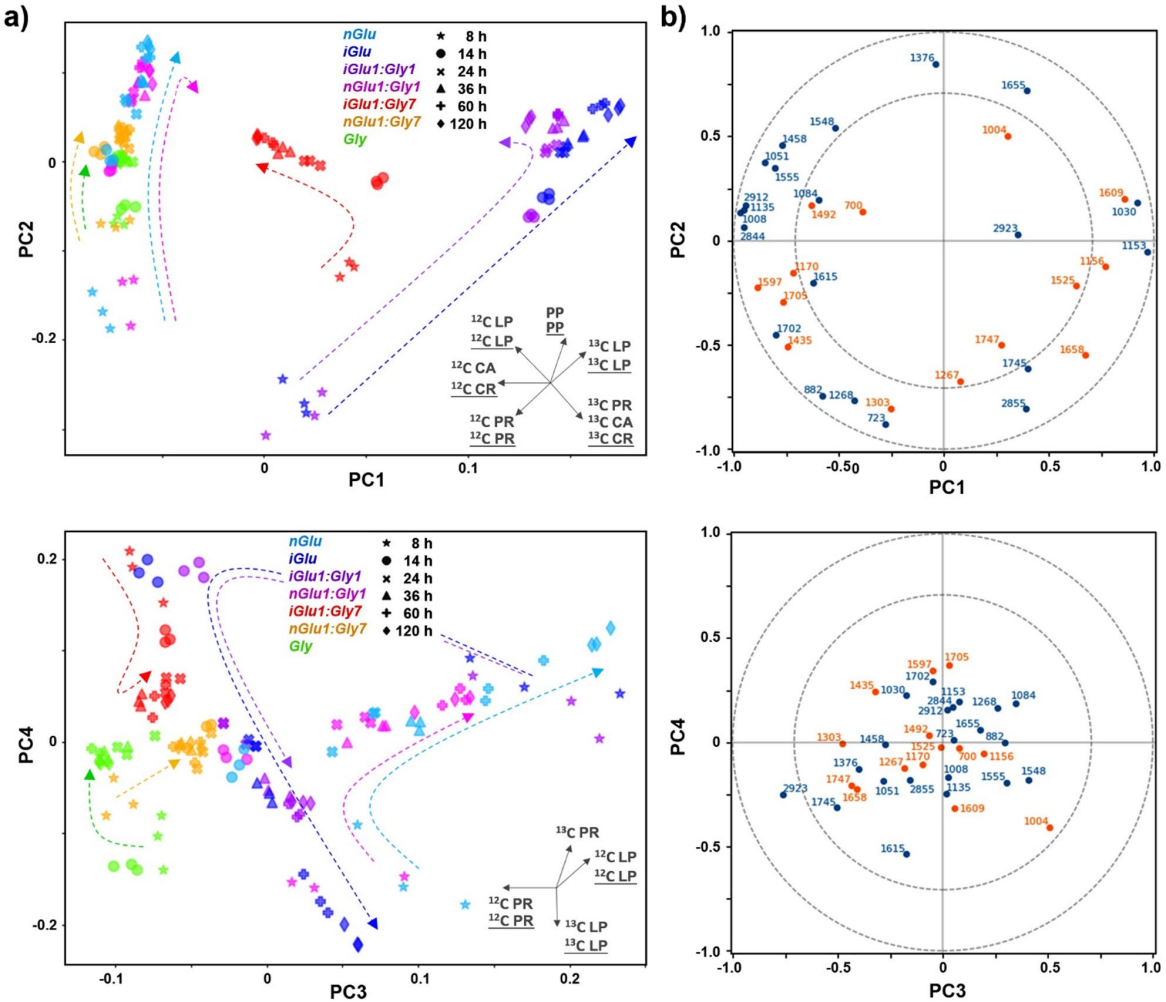
Multiblock consensus principal component analysis of HTS-FTIR and FT-Raman spectroscopic data. **a** Score plots of global scores of consensus principal component analysis (CPCA). The percent variances for the first five global PCs are 39.14, 23.44, 9.04, 4.31 and 2.36. The dashed lines represent approximations of temporal progression. The vectors are approximating the increase in the relative amount of metabolites: lipids (LP), proteins (PR), carbohydrates (CA), polyphosphates (PP), and carotenoids (CR); the vector information derived from FT-Raman data is *underlined* while the information derived from FTIR data is not underlined; ^12^C and ^13^C indicate carbon isotope composition. **b** CPCA correlation loading plots for the first four principal components. Correlation between FTIR (blue) and FT-Raman (orange). For the sake of clarity, only the selected variables are presented. Note that, as the spectral data for CPCA are in the second derivative form, high negative signal values correspond to high IR absorbance or Raman intensity

HTS-FTIR and FT-Raman spectral data blocks were analysed by consensus principal component analysis (CPCA) to assess and compare these two complementary vibrational methodologies for measurement of different chemical aspects of fungal biomass. Multiblock analysis offers a significant advantage over performing PCA on individual spectral data blocks of each spectroscopic method, as it reveals both common and method-specific analytical features more directly. In CPCA, spectral bands relevant to sample variation across blocks can be visualised in a common multiblock correlation loading plot. This allows for direct insights into similarities and differences between the two vibrational techniques. CPCA produces two types of scores: (1) global scores, which represent consensus variation between the two vibrational methods (Fig. 5a), and (2) block scores, which represent variation specific to each single data block (Figs S2 and S3 in the Supplementary Information). A high fraction of explained variance for a single block indicates that the global CPCA pattern effectively captures the variation within that block. The results show that the explained variance for the first five PCs is higher in the FTIR block (Fig. S2 in the Supplementary Information) compared to the FT-Raman block (Fig. S3 in the Supplementary Information), indicating that the variation in FTIR has a stronger covariance structure and is dominating the global CPCA pattern. At the early stage of cultivation, it is expected that the majority of the microbial biomass is derived from the yeast extract nutrient source. However, the global score plots show noticeable differences between the biomasses grown on isotopically labelled (^13^C) glucose and standard (^12^C) glucose as early as the 8-hour time point, indicating that glucose consumption has already begun by that time. After the initial divergence (0–14 h), the biomasses grown in mixed media with a large surplus of glycerol (*nGlu1:Gly7* and *iGlu1:Gly7*) show convergence in chemical composition after the 14-hour timepoint. The PCA score plots for individual blocks align with CPCA results and will not be discussed further.

Correlation loading plots (Fig. 5b) visualize the relationships between the global principal components and vibrational bands for both techniques in one plot. Characteristic peak wavenumbers well explained by CPCA components appear near the outer ring, where the outer and inner circles represent complete and 50% correlation, respectively. The correlation loading plot with PC1 and PC2 revealed the following connections between chemistry and wavenumbers: polyphosphates (Raman: 1170 cm^− 1^; IR: 1268, 882 cm^− 1^), ^12^C lipids (Raman: 1747, 1658 cm^− 1^; IR: 1745, 723 cm^− 1^), ^13^C lipids (Raman: 1705, 1597, 1435, 1303 cm^− 1^; IR: 1702 cm^− 1^), ^12^C proteins (Raman: 1004 cm^− 1^; IR: 1655 cm^− 1^), ^13^C proteins (IR: 1615 cm^− 1^), ^12^C carotenoids (Raman: 1525, 1156 cm^− 1^), ^13^C carotenoids (Raman: 1492 cm^− 1^), ^12^C carbohydrates (IR: 1153, 1030 cm^− 1^), ^13^C carbohydrates (IR: 1135, 1051, 1008 cm^− 1^). The correlation loading plot with PC3 and PC4 components showed the following connections: ^12^C lipids (Raman: 1747, 1658 cm^− 1^; IR: 1745 cm^− 1^), ^13^C lipids (Raman: 1705, 1597, 1435 cm^− 1^; IR: 1702 cm^− 1^), ^12^C proteins (Raman: 1004 cm^− 1^; IR: 1655, 1555 cm^− 1^), ^13^C proteins (IR: 1615 cm^− 1^). It is important to note that the assigned bands appear to be anticorrelated on the correlation loading plot. However, this is because the spectral data used for CPCA are in the second derivative form, where high negative signal values correspond to high IR absorbance or Raman intensity. Regarding the correlation loading plot with PC3 and PC4, the placement of almost all variables well within the inner circle in the plot indicates that none of the vibrational bands exhibit strong correlations with the principal components.

The chemical assignments of the vibrational bands derived from the correlation loading plots were used to visualize the chemical vector information presented in Fig. 5a, providing a detailed temporal representation of the chemical composition. The CPCA results clearly show a progression from protein-rich biomass in the initial phase (0–8 h) to modest carotenoid accumulation immediately following it (8–14 h), and finally to lipid-rich biomass in the later stages (24–120 h). It also shows how fungi grown in mixed carbon sources with a large surplus of glycerol and isotopically labelled (^13^C) glucose (*iGlu1:Gly7*) accumulate lipids and carotenoids during the first 14 h based on glucose and then switch to lipid accumulation based on glycerol afterward. This is in accordance with the published study that has shown that *M. circinelloides* is capable of utilizing both glycerol and glucose as carbon sources, with a clear preference for glucose [45]. In general, the CPCA highlights how Raman and IR are highly complementary techniques, providing mutually supportive insights that enhance the reliability and robustness of chemical band assignments.

### Raman microspectroscopy

The primary purpose of conducting measurements with Raman microspectroscopy was to obtain detailed information on carotenoid production, including their accumulation, molecular composition, and potential isotopic variations. The FT-Raman measurements with a 1064 nm excitation laser revealed the absence of the carotenoid-related band at 1525 cm⁻¹ in the spectra of fungi grown in mixed media with isotopically labelled (¹³C) glucose and with a large surplus of glycerol (*iGlu1:Gly7*). This strongly suggests that carotenoids are primarily derived from glucose rather than yeast extract or glycerol. To further investigate this finding, we conducted Raman measurements using a 785 nm excitation laser to assess whether the same observations are obtained under different excitation conditions. Compared to 1064 nm excitation laser, the resonant Raman effect for carotenoids is more pronounced with a 785 nm excitation laser because that wavelength is closer to the electronic absorption band of carotenoids, thus enhancing resonance and amplifying Raman scattering resulting with higher Raman intensities for carotenoid-related bands.

**Fig. 6:**
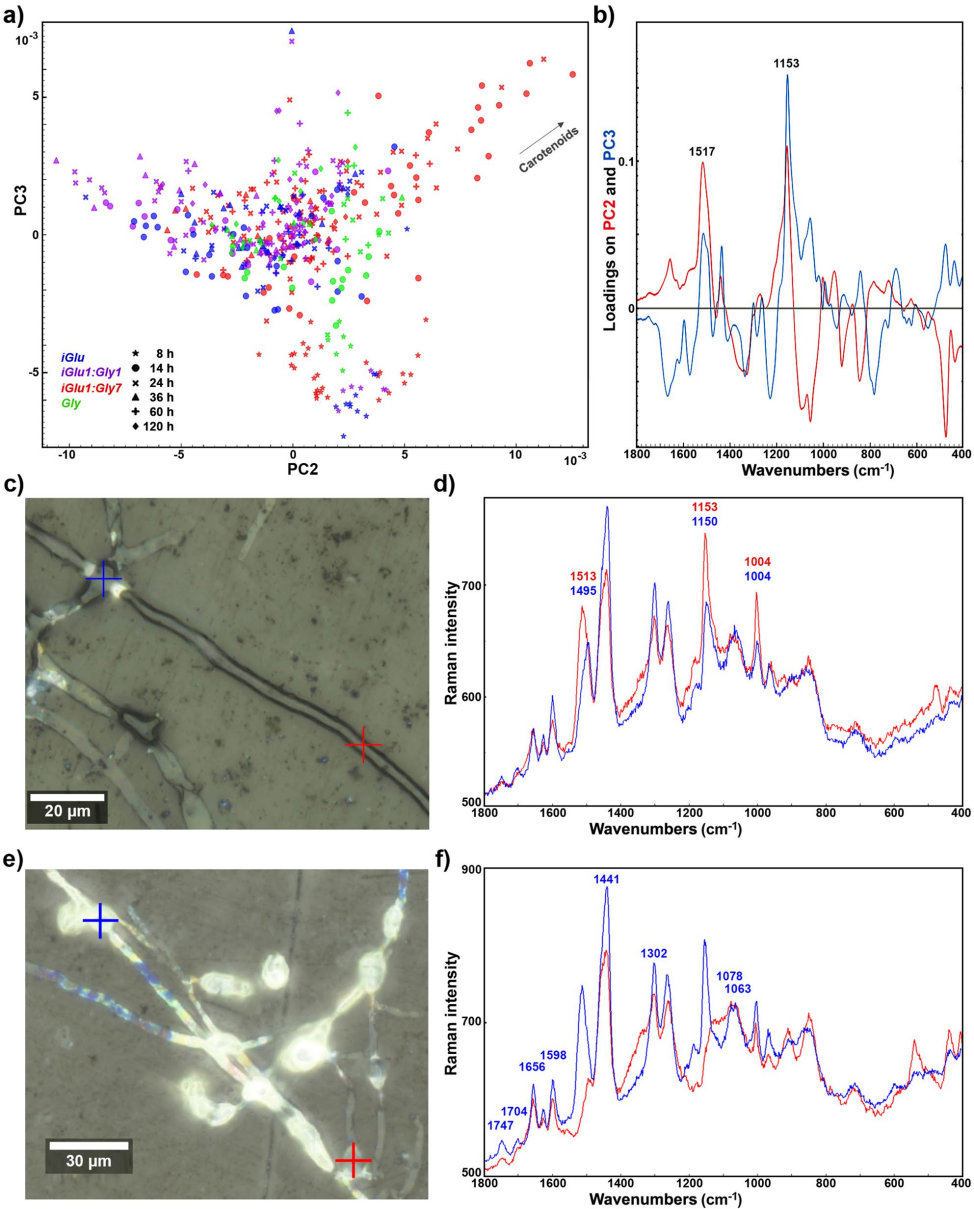
Raman microspectroscopy point measurement spectroscopic data. **a** PCA score plot of the second and third principal components. The percent variances for the first five global PCs are 49.87, 20.83, 10.40, 2.78 and 4.74. The vector is approximating the increase in the relative amount of carotenoids with mixed signals (ranging from 1525 to 1490 cm⁻¹). **b** PCA loadings plot on the second and third principal components. The data of the samples corresponding to nGlu, nGlu1:Gly1 and nGlu1:Gly7 media are omitted for clarity. **c** Brightfield light microscopy image of fungal tissue grown in mixed media with a large surplus of glycerol (iGlu1:Gly7) for 24h, with the designation of the two point-measurements (blue and red crosses). **d** The spectra corresponding to the two measurement positions displayed in (**c**) with labelled carotenoid-related bands. **e** Brightfield light microscopy image of fungal tissue grown in mixed media with a large surplus of glycerol (iGlu1:Gly7) for 60h, with the designation of the two point-measurements (blue and red crosses). **f** The spectra corresponding to the two measurement positions displayed in (**e**) with labelled lipid-related bands.

Figure 6. Raman microspectroscopy point measurement spectroscopic data. **a** PCA score plot of the second and third principal components. The percent variances for the first five global PCs are 49.87, 20.83, 10.40, 2.78 and 4.74. The vector is approximating the increase in the relative amount of carotenoids with mixed signals (ranging from 1525 to 1490 cm⁻¹). **b** PCA loadings plot on the second and third principal components. The data of the samples corresponding to *nGlu*, n*Glu1:Gly1* and n*Glu1:Gly7* media are omitted for clarity. **c** Brightfield light microscopy image of fungal tissue grown in mixed media with a large surplus of glycerol (*iGlu1:Gly7*) for 24 h, with the designation of the two point-measurements (blue and red crosses). **d** The spectra corresponding to the two measurement positions displayed in (c) with labelled carotenoid-related bands. **e** Brightfield light microscopy image of fungal tissue grown in mixed media with a large surplus of glycerol (*iGlu1:Gly7*) for 60 h, with the designation of the two point-measurements (blue and red crosses). **f** The spectra corresponding to the two measurement positions displayed in (**e**) with labelled lipid-related bands.

The PCA of the Raman microspectroscopy single-point measurement data revealed that the sample grown in mixed media with a large surplus of glycerol (*iGlu1:Gly7*) exhibits a different spectral pattern compared to the other samples, characterized by signals associated with carotenoid accumulation (Fig. 6). Although the carotenoid signals did not exhibit the typical wavenumbers associated with the ¹²C isotope (in particular, 1525 cm^− 1^), they also did not align with the wavenumbers characteristic of the ¹³C isotope (in particular, 1490 cm^− 1^). Remarkably, the carotenoid signals displayed variations in peak positions across wavenumbers, ranging from 1525 to 1490 cm^− 1^. Figure 6d shows an example where spectra with distinctly different carotenoid signals were obtained by measuring at two positions within a single hyphal structure, separated by just 80 μm, highlighting the localized variability in carotenoid composition at 14 h of cultivation. This strongly indicates that the carotenoids were assembled using two different carbon isotopes. The primary carotenoid in *Mucor circinelloides* is β-carotene, a molecule composed of 40 carbon atoms synthesized from eight isoprene units. In such a large molecule, there are numerous possible combinations of the two carbon isotopes within the molecular structure. These variations in isotope distribution can lead to slight shifts in vibrational modes, which, in turn, alter the positions and intensities of the carotenoid-related Raman peaks. While the source of the ^13^C isotope was obviously glucose, the source of ^12^C was most likely glycerol and not yeast extract. Yeast extract served as the primary nutrient source consumed during the early stages of microbial growth when the carotenoids are being produced, so its role in carotenoid production cannot be entirely excluded. However, if yeast extract serves as the secondary carbon source for biosynthesis of carotenoids it is unclear why the same type of mixed-carbon signals are not present in the spectra of *iGlu* sample. Interestingly, the biomass grown in mixed media containing an equal amount of glucose and glycerol (*iGlu1:Gly1*) does not exhibit the same type of mixed carotenoid signals (ranging from 1525 to 1490 cm⁻¹) as observed under *iGlu1:Gly7* conditions. This suggests that the utilization of glycerol for carotenoid synthesis occurs only under conditions of limited glucose availability, highlighting the influence of substrate composition on metabolic pathways. Figure 6f shows an example spectra of lipid bodies showing significant accumulation of lipids at 60 h of cultivation in iGlu1:Gly7 media. These lipids are predominantly based on glycerol and not glucose, as indicated by 12C=O stretching signals at 1747 cm-1, therefore corroborating the results of bulk biomass measurements with HTS techniques (Fig. 3).

**Fig. 7:**
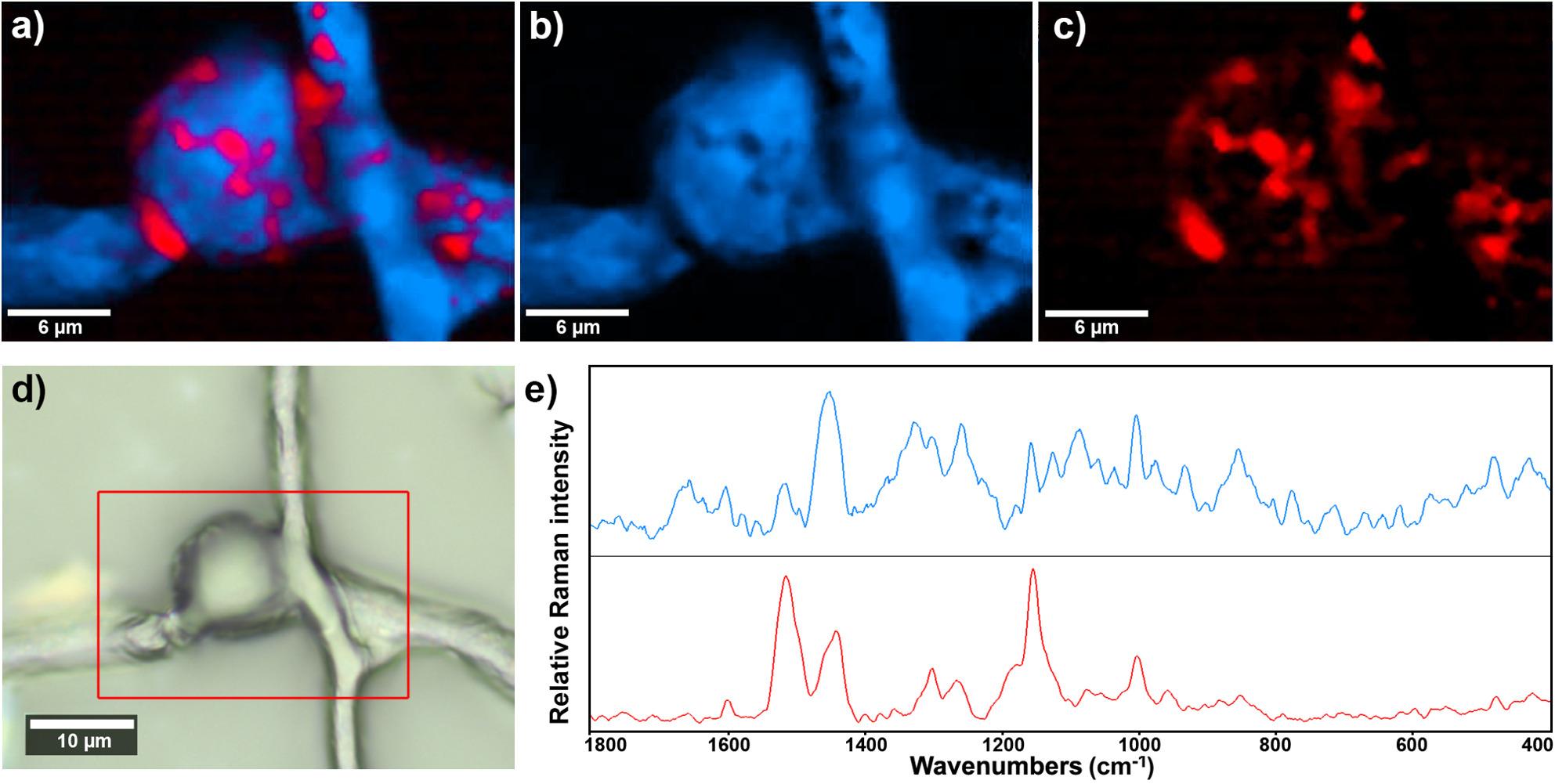
Basis analysis with two components of Raman microspectroscopy data of fungal tissue grown in mixed media with a large surplus of glycerol (iGlu1:Gly7) for 14h. **a** Resulting composite image of the fitting factors for the both components. Resulting image showing the fitting factors of (**b**) the first component corresponding to the protein- and carbohydrate-rich tissue, and **c** the second component corresponding to the carotenoid-rich tissue. **d** Brightfield light microscopy image of fungal tissue with the designation of the mapped Raman hyperspectral area (red rectangle). **e** The average spectra of the two components.

**Fig. 8:**
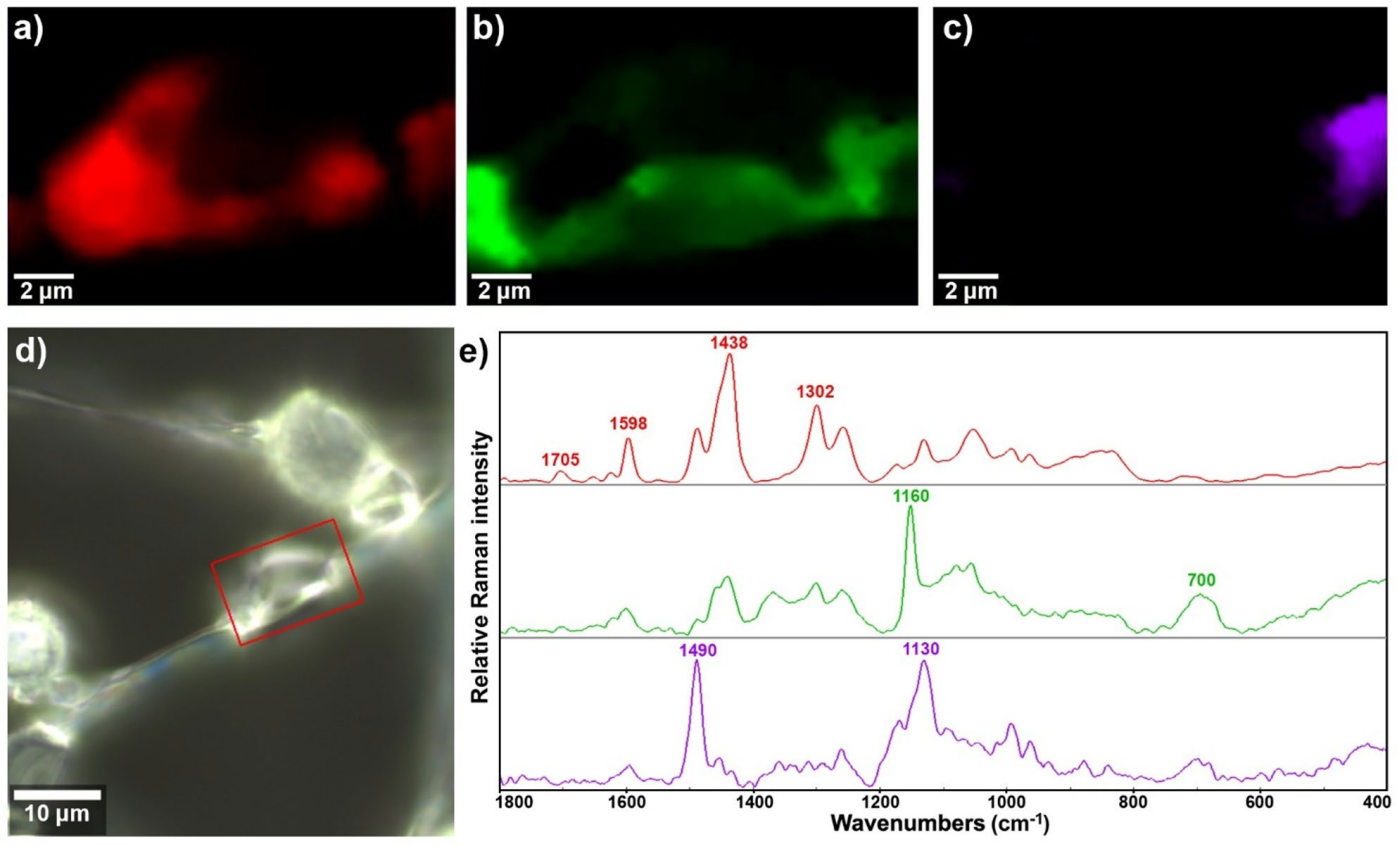
Basis analysis with three components of Raman microspectroscopy data of fungal tissue grown in mixed media with equal amount of glucose and glycerol (*iGlu1:Gly1*) for 60 h. Resulting images showing the fitting factors of: **a** the first component corresponding to the lipid- and carotenoid-rich tissue, **b** the second component corresponding to the polyphosphate-rich tissue, and **c** the third component corresponding to the carotenoid-rich tissue. **d** Light microscopy image of fungal tissue with the designation of the mapped Raman hyperspectral area (red rectangle). **e** The average spectra of the three components

Raman microspectroscopy imaging further reveals the complexity of distribution of carotenoids, lipids, and polyphosphates (Figs. 7 and 8), providing clear visualization of spatial domains associated with different metabolites. This technique offers enhanced sensitivity to spatial variations. In *iGlu1:Gly7* sample, the signals related to carotenoids are particularly broad, likely reflecting the isotopic heterogeneity within the carotenoid molecules (Fig. 7). Raman microspectroscopy imaging further revealed the spatial organization of phosphate and lipid structural domains, providing clear visualization of their distinct distributions within the fungal biomass (Fig. 8). The findings demonstrate how this technique could provide valuable data for future studies to investigate the interaction between phosphorus-based and carbon-based energy storage mechanisms in filamentous fungi grown on mixed carbon sources. In general, the results of FT-Raman (with 1064 nm excitation laser) and Raman microspectroscopy (with 785 nm excitation laser) underscore the importance of using complementary Raman techniques. This is consistent with previous studies, which highlight that different excitation laser wavelengths and measurement modalities each offer distinct advantages and limitations [63].

### FTIR microspectroscopy

**Fig. 9:**
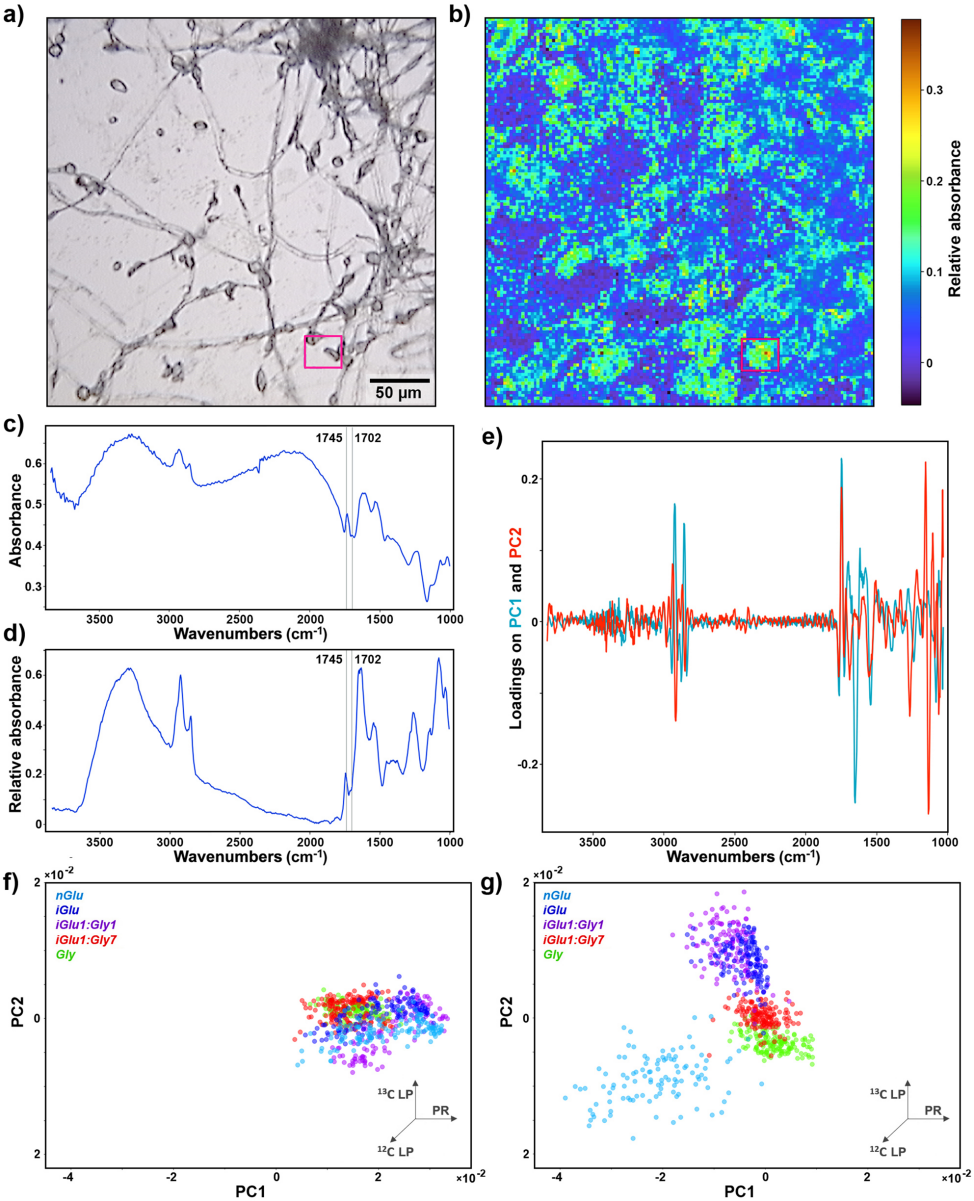
FTIR microspectroscopy imaging data. **a** Brightfield light microscopy image of fungal tissue grown in mixed media with a large surplus of glycerol (*iGlu1:Gly7*) for 120 h with the designation of the spectral averaging area (magenta rectangle). **b** Hyperspectral image (corresponding to the light microscopy image) generated by preprocessing the spectral data and plotting relative absorbance values at 1745 cm^− 1^. Average spectra obtained by averaging all **c** raw spectra and **d** preprocessed spectra in the area labelled in (**a**) and (**b**). **e** PCA of binned images with loadings plot of the first two principal components. The percent variances for the first five PCs are 46.62, 20.62, 10.82, 6.23 and 3.04. PCA scores plots for spectra of the samples grown for **f** 8 h and **g** 36 h; For clarity, only data from two timepoints are depicted and the datapoints of the samples corresponding to n*Glu1:Gly1* and n*Glu1:Gly7* media are omitted; The common PCA space was used to plot both PCA scores; The vectors are approximating the increase in the relative amount of lipids (LP) and proteins (PR) (^12^C and ^13^C indicate carbon isotope composition)

The primary purpose of conducting FTIR microspectroscopy imaging was to obtain spatial information on lipid production and storage at cellular level. The FTIR hyperspectral image of the biomasses grown in mixed media with a large surplus of glycerol (*iGlu1:Gly7*) reveals sparse spatial domains with a higher-than-average lipid content (Fig. 9a and b). These lipids are produced from glycerol as is clearly evidenced by the TAG-related ^12^C = O stretching vibration at 1745 cm^− 1^ observed in both the raw and preprocessed spectra (Fig. 9c and d).

The PCA of the hyperspectral data reveals that, at the early stage of fermentation (after 8 h), all samples grown in different media exhibit a relatively uniform chemical composition of biomass, dominated by proteins (Fig. 9e-g). However, after 36 h of fermentation, a clear distinction emerges between lipid-rich biomass grown on ^12^C glucose and ^13^C glucose. Among the samples grown on mixed carbon source media, the biomass from the medium containing equal amounts of glucose and glycerol (*iGlu1:Gly1*) is extremely similar to the biomass grown in pure ^13^C glucose medium. In contrast, the biomass from the mixed carbon source medium with a large surplus of glycerol (*iGlu1:Gly7*) predominantly exhibits a chemical composition similar to biomass grown in glycerol. However, some parts of *iGlu1:Gly7* biomass also exhibit chemical similarities with the biomass grown in pure ^12^C glucose medium. This corroborates the observations that a modest amount of lipids is being synthesized from glycerol.

We have recently presented and validated a deep learning-based calibration transfer method that enables quantitative chemical analysis in FTIR microspectroscopic imaging of oleaginous filamentous fungi, including *M. circinelloides*, by adapting regression models for glucosamine and lipid (TAG) content from bulk measurements [22]. This allows for obtaining the spatial distribution of lipid and glucosamine (i.e. chitin and chitosan) content in intact fungal samples in a nondestructive and label-free manner. This approach, when combined with stable isotope labelling, could provide spatially resolved quantitative tracking of carbon flow and metabolite distribution in complex fermentation systems. In the future, such a combined strategy has the potential to reveal new insights into the metabolic dynamics of oleaginous fungi grown on mixed substrates.

### The timeline of carbon utilisation

As a consensus of all the measurements, the following timeline for the utilization of different carbon sources can be constructed. At the early stage of fermentation (the first 8 h), the biomass is rich in proteins and carbohydrates. This is primarily attributed to the utilization of yeast extract, although some glucose utilization (if available in the growth media) is also observed. In the next phase of fermentation (from 8 to 14 h), the biomass begins to show signs of polyphosphate, lipid and carotenoid accumulation. This phase represents the principal stage for carotenoid accumulation, and in *iGlu1:Gly7* media the carotenoids are assembled from mixed sources, most likely glucose and glycerol, though contribution of yeast extract cannot be excluded. The lipid accumulation, primarily in the form of TAGs, is largely attributed to the utilization of glucose. Consequently, the fermentations in media deficient in glucose result in biomasses with little to no accumulated TAGs. In the subsequent phases (beyond 14 h), the biomass continues to accumulate polyphosphates. Moreover, lipids will accumulate in large amounts if significant amount of glucose is present in the medium. In mixed carbon source media with a large surplus of glycerol, only modest amounts of lipids will be accumulated, while in glycerol-only media, lipid accumulation will be negligible.

For obtaining information on the timeline of carbon utilisation one can imagine supplementing the vibrational spectroscopy methods with other analytical techniques to gain a more comprehensive understanding. Direct measurement of glucose and glycerol consumption, for example, through HPLC analysis, could serve as a valuable supplement to the spectroscopy findings by providing quantitative data on substrate utilization. However, conducting these measurements could present significant challenges due to the limited amount of media available, as the small scale of cultivation is constrained by the high costs associated with the isotope-labelled substrates. For this very reason, we chose not to conduct measurements of glucose and glycerol consumption in this study. Interestingly, FT-Raman spectroscopy could also be employed to measure glucose and glycerol concentrations in the media, as we have demonstrated recently for cultivations of yeasts and thraustochytrids [13]. Unfortunately, those predictive models were developed after the completion of this study and were therefore not available to us at the time of this work.

## Conclusions

Multimodal vibrational spectroscopy, utilizing both FTIR and FT-Raman for bulk biomass measurements and microspectroscopy for imaging, is a powerful tool for detailed molecular analysis. It leverages the strengths of each technique to deliver comprehensive insights into the vibrational properties, structure, and composition of microbial biomass. The main goal of the study was to evaluate stable isotope labelling combined with infrared and Raman spectroscopies as tools for studying carbon utilization in fermentations with mixed carbon substrates. In this regard, the study was a complete success, as we were able to identify the flux of mixed carbon sources to exact metabolites. Compared to fermentation conducted in glycerol media, fermentations in mixed carbon source media with a large surplus of glycerol resulted in slightly improved glycerol utilization for biomass production and lipid accumulation. Glycerol consumption can be estimated based on the ^12^C lipid signals, as glycerol was the sole source of ^12^C when ^13^C isotope-labelled glucose was present in the media. Comparing the ^12^C lipid signals between the mixed substrate and the glycerol-only substrate, higher signal intensity was observed in the biomass grown on the mixed substrate. This was particularly evident in the lipid-related ^12^C = O stretching signals detected by infrared and Raman spectroscopies. For example, the FTIR microspectroscopy imaging of biomass grown in mixed media with a large surplus of glycerol reveals sparse spatial domains with elevated ^12^C lipid content.

In general, only modest co-utilization of glucose and glycerol was achieved, which is expected based on the previously published studies. It is important to note that, considering the main goal of the study, optimizing co-utilization was beyond the scope of this study. The study has also revealed the importance of polyphosphates as vital additional energy reserve molecules, accompanying triglyceride lipids. Future research on mixed carbon substrates may study in detail phosphorus-based energy reserves, particularly polyphosphate accumulation. This could be effectively explored by limiting phosphorus content in the medium, as demonstrated in our previous studies [15, 16, 18, 42].

The use of different IR and Raman techniques in various settings provided complementary chemical information. Although the study involved drying and, in the case of HTS-FTIR, also disrupting the biomass for analysis, the approach demonstrates potential for non-invasive measurements, particularly through the use of Raman spectroscopy. This highlights its suitability for real-time, in situ monitoring without the need for extensive sample preparation. Moreover, the results highlight the critical importance of employing complementary Raman techniques to obtain a more comprehensive understanding of molecular composition and isotopic distribution. For example, Raman microspectroscopy imaging visualized the distinct spatial distributions of phosphate and lipid domains within the fungal biomass, as well as the isotopic heterogeneity of carotenoids. Different excitation wavelengths and Raman modalities can provide unique insights, as they may enhance or suppress specific vibrational modes, enabling the detection of subtle spectral features that might otherwise be overlooked. In general, the multi-modal approach ensures a more robust and reliable interpretation of the data. This enabled us to link the two carbon sources (glucose and glycerol) to distinct metabolites and to obtain the detailed timeline of metabolite production. The developed methodology is broadly applicable to the low-volume screening and optimization stages of bioprocesses development. Isotopic labelling of a selected carbon source enables discrimination of substrate utilization sequences and metabolic fates, thereby supporting rational optimization of substrate concentrations and feeding strategies.

## Supplementary Information

Below is the link to the electronic supplementary material.

Supplementary Material 1

## Data Availability

All datasets generated for this study are available in the Zenodo repository upon reasonable request from the corresponding author: DOI 10.5281/zenodo.17414246.

## References

[1] Liu N, Santala S, Stephanopoulos G. Mixed carbon substrates: a necessary nuisance or a missed opportunity? Curr Opin Biotechnol. 2020;62:15–21. 31513988 10.1016/j.copbio.2019.07.003

[2] Dzurendova S, Losada CB, Dupuy-Galet BX, Fjær K, Shapaval V. Mucoromycota fungi as powerful cell factories for modern biorefinery. Appl Microbiol Biotechnol. 2022;106:101–15. 34889982 10.1007/s00253-021-11720-1

[3] Liu YS, Tang YH, Gao HY, Zhang WM, Jiang YJ, Xin FX, Jiang M. Challenges and Future Perspectives of Promising Biotechnologies for Lignocellulosic Biorefinery. Molecules 2021, 26. 10.3390/molecules26175411 PMC8433869 34500844

[4] Chozhavendhan S, Kumar RP, Elavazhagan S, Barathiraja B, Jayakumar M, Varjani SJ. Utilization of Crude Glycerol from Biodiesel Industry for the Production of Value-Added Bioproducts. Waste Wealth 2018:65–82.

[5] Papanikolaou S, Rontou M, Belka A, Athenaki M, Gardeli C, Mallouchos A, Kalantzi O, Koutinas AA, Kookos IK, Zeng AP, Aggelis G. Conversion of biodiesel-derived glycerol into biotechnological products of industrial significance by yeast and fungal strains. Eng Life Sci. 2017;17:262–81. 32624773 10.1002/elsc.201500191 PMC6999228

[6] Cavka A, Jönsson LJ. Comparison of the growth of filamentous fungi and yeasts in lignocellulose-derived media. Biocatal Agric Biotechnol. 2014;3:197–204.

[7] Wu Y, Shen X, Yuan Q, Yan Y. Metabolic Engineering Strategies for Co-Utilization of Carbon Sources in Microbes. In *Bioengineering*, vol. 32016. 10.3390/bioengineering3010010 PMC5597168 28952572

[8] An N, Chen X, Sheng HK, Wang J, Sun XX, Yan YJ, Shen XL, Yuan QP. Rewiring the microbial metabolic network for efficient utilization of mixed carbon sources. J Ind Microbiol Biotechnol 2021, 48. 10.1093/jimb/kuab040 PMC8788776 34215883

[9] Schalk R, Braun F, Frank R, Rädle M, Gretz N, Methner FJ, Beuermann T. Non-contact Raman spectroscopy for in-line monitoring of glucose and ethanol during yeast fermentations. Bioprocess Biosyst Eng. 2017;40:1519–27. 28656375 10.1007/s00449-017-1808-9

[10] Wang QY, Li ZG, Ma ZH, Liang LQ. Real time monitoring of multiple components in wine fermentation using an on-line auto-calibration Raman spectroscopy. Sens Actuators B-Chemical. 2014;202:426–32.

[11] Wieland K, Masri M, von Poschinger J, Brück T, Haisch C. Non-invasive Raman spectroscopy for time-resolved in-line lipidomics. RSC Adv. 2021;11:28565–72. 35478569 10.1039/d1ra04254h PMC9038134

[12] Akulava V, Tafintseva V, Blazhko U, Kohler A, Miamin U, Valentovich L, Shapaval V. Global biochemical profiling of fast-growing Antarctic bacteria isolated from meltwater ponds by high-throughput FTIR spectroscopy. PLoS ONE 2024, 19. 10.1371/journal.pone.0303298 PMC11182503 38885224

[13] Dzurendova S, Olsen PM, Byrtusova D, Tafintseva V, Shapaval V, Horn SJ, Kohler A, Szotkowski M, Marova I, Zimmermann B. Raman spectroscopy online monitoring of biomass production, intracellular metabolites and carbon substrates during submerged fermentation of oleaginous and carotenogenic microorganisms. Microb Cell Fact 2023, 22. 10.1186/s12934-023-02268-y PMC10729511 38110983

[14] Forfang K, Zimmermann B, Kosa G, Kohler A, Shapaval V. FTIR Spectroscopy for Evaluation and Monitoring of Lipid Extraction Efficiency for Oleaginous Fungi. PLoS ONE 2017, 12. 10.1371/journal.pone.0170611 PMC5261814 28118388

[15] Dzurendova S, Shapaval V, Tafintseva V, Kohler A, Byrtusova D, Szotkowski M, Marova I, Zimmermann B. Assessment of Biotechnologically Important Filamentous Fungal Biomass by Fourier Transform Raman Spectroscopy. Int J Mol Sci 2021, 22. 10.3390/ijms22136710 PMC8269384 34201486

[16] Dzurendova S, Zimmermann B, Kohler A, Reitzel K, Nielsen UG, Dupuy–Galet BX, Leivers S, Horn SJ, Shapaval V. Calcium Affects Polyphosphate and Lipid Accumulation in Mucoromycota Fungi. J Fungi 2021, 7. 10.3390/jof7040300 PMC8071181 33920847

[17] Dzurendova S, Zimmermann B, Kohler A, Tafintseva V, Slany O, Certik M, Shapaval V. Microcultivation and FTIR spectroscopy-based screening revealed a nutrient-induced co-production of high-value metabolites in oleaginous Mucoromycota fungi. PLoS ONE. 2020;15:e0234870. 32569317 10.1371/journal.pone.0234870 PMC7307774

[18] Dzurendova S, Zimmermann B, Tafintseva V, Kohler A, Horn SJ, Shapaval V. Metal and Phosphate Ions Show Remarkable Influence on the Biomass Production and Lipid Accumulation in Oleaginous Mucor circinelloides. J Fungi (Basel) 2020, 6. 10.3390/jof6040260 PMC7711463 33143254

[19] Kosa G, Kohler A, Tafintseva V, Zimmermann B, Forfang K, Afseth NK, Tzimorotas D, Vuoristo KS, Horn SJ, Mounier J, Shapaval V. Microtiter plate cultivation of oleaginous fungi and monitoring of lipogenesis by high-throughput FTIR spectroscopy. Microb Cell Fact 2017, 16. 10.1186/s12934-017-0716-7 PMC5466753 28599651

[20] Kosa G, Shapaval V, Kohler A, Zimmermann B. FTIR spectroscopy as a unified method for simultaneous analysis of intra- and extracellular metabolites in high-throughput screening of microbial bioprocesses. Microb Cell Fact 2017, 16. 10.1186/s12934-017-0817-3 PMC5683213 29132358

[21] Losada CB, Slany O, Byrtusová D, Zimmermann B, Horn SJ, Kohler A, Shapaval V. Compatible traits of oleaginous Mucoromycota fungi for lignocellulose-based simultaneous saccharification and fermentation. Biotechnol Biofuels Bioprod 2025, 18. 10.1186/s13068-025-02621-w PMC11854021 39994750

[22] Magnussen EA, Zimmermann B, Dzurendova S, Slany O, Tafintseva V, Liland KH, To̷ndel K, Shapaval V, Kohler A. Calibration for Quantitative Chemical Analysis in IR Microscopic Imaging. Anal Chem. 2025;97:21947–55. 41050996 10.1021/acs.analchem.5c03049 PMC12529472

[23] Shapaval V, Deniset-Besseau A, Dubava D, Dzurendova S, Heitmann Solheim J, Kohler A. Multiscale spectroscopic analysis of lipids in dimorphic and oleaginous Mucor circinelloides accommodate sustainable targeted lipid production. Fungal Biology Biotechnol. 2023;10:2. 10.1186/s40694-023-00148-z PMC9843973 36647105

[24] Blazhko U, Byrtusová D, Shapaval V, Kohler A, Sandt C, Zimmermann B. Submicron infrared spectroscopy assessment of single-cell phenotypic diversity in microbial lipid production. Microb Cell Fact. 2025;24:171. 40685363 10.1186/s12934-025-02794-x PMC12278496

[25] Wang Y, Huang WE, Cui L, Wagner M. Single cell stable isotope probing in microbiology using Raman microspectroscopy. Curr Opin Biotechnol. 2016;41:34–42. 27149160 10.1016/j.copbio.2016.04.018

[26] Shams S, Ahmed S, Smaje D, Tengsuttiwat T, Lima C, Goodacre R, Muhamadali H. Application of infrared spectroscopy to study carbon-deuterium kinetics and isotopic spectral shifts at the single-cell level. Spectrochimica Acta Part a-Molecular Biomol Spectrosc 2025, 327. 10.1016/j.saa.2024.125374 39522229

[27] Venkata HNN, Shigeto S. Stable Isotope-Labeled Raman Imaging Reveals Dynamic Proteome Localization to Lipid Droplets in Single Fission Yeast Cells. Chem Biol. 2012;19:1373–80. 23177192 10.1016/j.chembiol.2012.08.020

[28] Noothalapati H, Shigeto S. Exploring Metabolic Pathways in Vivo by a Combined Approach of Mixed Stable Isotope-Labeled Raman Microspectroscopy and Multivariate Curve Resolution Analysis. Anal Chem. 2014;86:7828–34. 24975289 10.1021/ac501735c

[29] Huang WE, Griffiths RI, Thompson IP, Bailey MJ, Whiteley AS. Raman microscopic analysis of single microbial cells. Anal Chem. 2004;76:4452–8. 15283587 10.1021/ac049753k

[30] Huang WE, Stoecker K, Griffiths R, Newbold L, Daims H, Whiteley AS, Wagner M. Raman-FISH: combining stable-isotope Raman spectroscopy and fluorescence hybridization for the single cell analysis of identity and function. Environ Microbiol. 2007;9:1878–89. 17635536 10.1111/j.1462-2920.2007.01352.x

[31] Li MQ, Canniffe DP, Jackson PJ, Davison PA, FitzGerald S, Dickman MJ, Burgess JG, Hunter CN, Huang WE. Rapid resonance Raman microspectroscopy to probe carbon dioxide fixation by single cells in microbial communities. ISME J. 2012;6:875–85. 22113377 10.1038/ismej.2011.150 PMC3309358

[32] Muhamadali H, Chisanga M, Subaihi A, Goodacre R. Combining Raman and FT-IR Spectroscopy with Quantitative Isotopic Labeling for Differentiation of E. coli Cells at Community and Single Cell Levels. Anal Chem. 2015;87:4578–86. 25831066 10.1021/acs.analchem.5b00892

[33] Kubryk P, Kölschbach JS, Marozava S, Lueders T, Meckenstock RU, Niessner R, Ivleva NP. Exploring the Potential of Stable Isotope (Resonance) Raman Microspectroscopy and Surface-Enhanced Raman Scattering for the Analysis of Microorganisms at Single Cell Level. Anal Chem. 2015;87:6622–30. 26010835 10.1021/acs.analchem.5b00673

[34] Lima C, Muhamadali H, Goodacre R. Simultaneous Raman and Infrared Spectroscopy of Stable Isotope Labelled. Sensors 2022, 22. 10.3390/s22103928 PMC9145054 35632337

[35] Burr DJ, Drauschke J, Kanevche K, Kümmel S, Stryhanyuk H, Heberle J, Perfumo A, Elsaesser A. Stable Isotope Probing-nanoFTIR for Quantitation of Cellular Metabolism and Observation of Growth-Dependent Spectral Features. Small 2024, 20. 10.1002/smll.202400289 38708804

[36] Karlo J, Dhillon AK, Siddhanta S, Singh SP. Reverse stable isotope labelling with Raman spectroscopy for microbial proteomics. J Biophotonics 2024, 17. 10.1002/jbio.202300341 38010366

[37] Sun YF, Li SS, Si Y, Niu YF, Yang JZ, Liu YH, Dong L, Zhu PF, Dai J, Yang F. Dual-Stable-Isotope-Probed Raman microspectroscopy reveals the metabolic dynamic of Streptococcus mutans. Spectrochimica Acta Part a-Molecular Biomol Spectrosc 2024, 304. 10.1016/j.saa.2023.123318 37703791

[38] Taylor GT, Suter EA, Li ZQ, Chow S, Stinton D, Zaliznyak T, Beaupré SR. Single-Cell Growth Rates in Photoautotrophic Populations Measured by Stable Isotope Probing and Resonance Raman Microspectrometry. Front Microbiol 2017, 8. 10.3389/fmicb.2017.01449 PMC5541042 28824580

[39] Weber F, Zaliznyak T, Edgcomb VP, Taylor GT. Using Stable Isotope Probing and Raman Microspectroscopy To Measure Growth Rates of Heterotrophic Bacteria. Appl Environ Microbiol 2021, 87. 10.1128/AEM.01460-21 PMC8579647 34495689

[40] Yasuda M, Takeshita N, Shigeto S. Deuterium-labeled Raman tracking of glucose accumulation and protein metabolic dynamics in Aspergillus nidulans hyphal tips. Sci Rep 2021, 11. 10.1038/s41598-020-80270-9 PMC7809412 33446770

[41] Fung AA, Shi LY. Mammalian cell and tissue imaging using Raman and coherent Raman microscopy. Wiley Interdisciplinary Reviews-Systems Biology Med 2020, 12. 10.1002/wsbm.1501 PMC7554227 32686297

[42] Dzurendova S, Zimmermann B, Tafintseva V, Kohler A, Ekeberg D, Shapaval V. The influence of phosphorus source and the nature of nitrogen substrate on the biomass production and lipid accumulation in oleaginous Mucoromycota fungi. Appl Microbiol Biotechnol. 2020;104:8065–76. 32789746 10.1007/s00253-020-10821-7 PMC7447667

[43] Losada CB, Di Bartolomeo F, Wentzel A, Markussen S, Dzurendova S, Zimmermann B, Fjaer K, Slany O, Várnai A, Hansen LD, et al. Simultaneous production of fatty acids and amino polysaccharides from Norway spruce hydrolysates using oleaginous Mucor circinelloides. Sci Rep. 2025;15:14106. 40269125 10.1038/s41598-025-98549-0 PMC12019349

[44] Pawlowska J, Okrasinska A, Kislo K, Aleksandrzak-Piekarczyk T, Szatraj K, Dolatabadi S, Muszewska A. Carbon assimilation profiles of mucoralean fungi show their metabolic versatility. Sci Rep 2019, 9. 10.1038/s41598-019-48296-w PMC6694110 31413281

[45] Morales RLC, Castellanos AD, Zazueta-Sandoval R. Analysis of glycerol dehydrogenase activities present in Mucor circinelloides YR-1. Antonie Van Leeuwenhoek Int J Gen Mol Microbiol. 2010;98:437–45. 10.1007/s10482-010-9457-x 20512634

[46] Kosa G, Zimmermann B, Kohler A, Ekeberg D, Afseth NK, Mounier J, Shapaval V. High-throughput screening of Mucoromycota fungi for production of low- and high-value lipids. Biotechnol Biofuels 2018, 11. 10.1186/s13068-018-1070-7 PMC5851148 29563969

[47] Kohler A, Solheim JH, Tafintseva V, Zimmermann B, Shapaval V. 3.03 - Model-Based Pre-Processing in Vibrational Spectroscopy. In *Comprehensive Chemometrics (Second Edition).* Edited by Brown S, Tauler R, Walczak B. Oxford: Elsevier; 2020: 83–100.

[48] Demsar J, Curk T, Erjavec A, Gorup C, Hocevar T, Milutinovic M, Mozina M, Polajnar M, Toplak M, Staric A, et al. Orange: Data Mining Toolbox in Python. J Mach Learn Res. 2013;14:2349–53.

[49] Toplak M, Birarda G, Read S, Sandt C, Rosendahl SM, Vaccari L, Demšar J, Borondics F. Infrared Orange: Connecting Hyperspectral Data with Machine Learning. Synchrotron Radiation News. 2017;30:40–5.

[50] Magnussen EA, Solheim JH, Blazhko U, Tafintseva V, Tondel K, Liland KH, Dzurendova S, Shapaval V, Sandt C, Borondics F, Kohler A. Deep convolutional neural network recovers pure absorbance spectra from highly scatter-distorted spectra of cells. J Biophotonics 2020, 13. 10.1002/jbio.202000204 32844585

[51] Magnussen EA, Zimmermann B, Blazhko U, Dzurendova S, Dupuy–Galet B, Byrtusova D, Muthreich F, Tafintseva V, Liland KH, Tøndel K, et al. Deep learning-enabled Inference of 3D molecular absorption distribution of biological cells from IR spectra. Commun Chem. 2022;5:175. 36697906 10.1038/s42004-022-00792-3 PMC9814771

[52] Muthreich F, Magnussen EA, Solheim JH, Tafintseva V, Kohler A, Seddon AWR, Zimmermann B. Analytical and experimental solutions for Fourier transform infrared microspectroscopy measurements of microparticles: A case study on Quercus pollen. Anal Chim Acta 2025, 1351. 10.1016/j.aca.2025.343879 40187871

[53] Tafintseva V, Shapaval V, Smirnova M, Kohler A. Extended multiplicative signal correction for FTIR spectral quality test and pre-processing of infrared imaging data. J Biophotonics 2020, 13. 10.1002/jbio.201960112 31793214

[54] Guo SX, Kohler A, Zimmermann B, Heinke R, Stockel S, Rosch P, Popp J, Bocklitz T. Extended Multiplicative Signal Correction Based Model Transfer for Raman Spectroscopy in Biological Applications. Anal Chem. 2018;90:9787–95. 30016081 10.1021/acs.analchem.8b01536

[55] Zimmermann B, Kohler A. Optimizing Savitzky-Golay Parameters for Improving Spectral Resolution and Quantification in Infrared Spectroscopy. Appl Spectrosc. 2013;67:892–902. 23876728 10.1366/12-06723

[56] Hassani S, Martens H, Qannari EM, Hanafi M, Borge GI, Kohler A. Analysis of -omics data: Graphical interpretation- and validation tools in multi-block methods. Chemometr Intell Lab Syst. 2010;104:140–53.

[57] Westerhuis JA, Kourti T, MacGregor JF. Analysis of multiblock and hierarchical PCA and PLS models. J Chemom. 1998;12:301–21.

[58] Schmidt U, Hild S, Ibach W, Hollricher O. Characterization of thin polymer films on the nanometer scale with Confocal Raman AFM. Macromolecular Symposia. 2005;230:133–43.

[59] Kerkaert JD, Huberman LB. Regulation of nutrient utilization in filamentous fungi. Appl Microbiol Biotechnol. 2023;107:5873–98. 37540250 10.1007/s00253-023-12680-4 PMC10983054

[60] Werner TP, Amrhein N, Freimoser FM. Specific localization of inorganic polyphosphate (poly P) in fungal cell walls by selective extraction and immunohistochemistry. Fungal Genet Biol. 2007;44:845–52. 17320430 10.1016/j.fgb.2007.01.008

[61] Gray MJ, Jakob U. Oxidative stress protection by polyphosphate - new roles for an old player. Curr Opin Microbiol. 2015;24:1–6. 25589044 10.1016/j.mib.2014.12.004 PMC4380828

[62] Naz T, Nazir Y, Nosheen S, Ullah S, Halim H, Fazili AA, Li SQ, Mustafa K, Mohamed H, Yang W, Song YD. Redirecting Metabolic Flux towards the Mevalonate Pathway for Enhanced β-Carotene Production in M. circinelloides CBS 277.49. *Biomed Research International* 2020, 2020. 10.1155/2020/8890269 PMC7785371 33457420

[63] Salbreiter M, Frempong SB, Even S, Wagenhaus A, Girnus S, Rösch P, Popp J. Lighting the Path: Raman Spectroscopy’s Journey Through the Microbial Maze. Molecules 2024, 29. 10.3390/molecules29245956 PMC11870064 39770046

