# Supplementary material for: Vibrational spectroscopy methodology for profiling of mixed carbon metabolism in carotenogenic and oleaginous Mucor circinelloides
Source: Microb Cell Fact. 2026 Feb 21;25:80. doi: 10.1186/s12934-026-02963-6 (PMC13032529; doi:10.1186/s12934-026-02963-6)
Supplement: Supplementary file 1 — Supplementary Material 1 [file 12934_2026_2963_MOESM1_ESM.pdf]

Supplementary Material

Vibrational spectroscopy profiling of mixed carbon metabolism in carotenogenic and oleaginous *Mucor circinelloides*

Simona Dzurendová[1,2], Eirik Almklov Magnussen[1], Volha Shapaval[1], Achim Kohler[1] and Boris Zimmermann[1]*

[1]Faculty of Science and Technology, Norwegian University of Life Sciences, Ås, Norway
[2]Faculty of Chemistry, Brno University of Technology, Brno, Czechia

Correspondence address: Faculty of Science and Technology, Norwegian University of Life Sciences, Postbox 5003, 1432 Ås, Norway

*Corresponding author:
Boris Zimmermann
Faculty of Science and Technology
Norwegian University of Life Sciences
Drøbakveien 31, 1432 Ås, Norway.
Tel: +47 6723 1576
Fax: +47 6496 5001
E-mail: boris.zimmermann@nmbu.no

Table of Contents **Page**

Figure S1. Estimates of total lipids and total phosphorus S-2
Figure S2. CPCA score and loading plots for HTS-FTIR individual blocks S-3
Figure S3. CPCA score and loading plots for HTS-FT-Raman individual blocks S-4

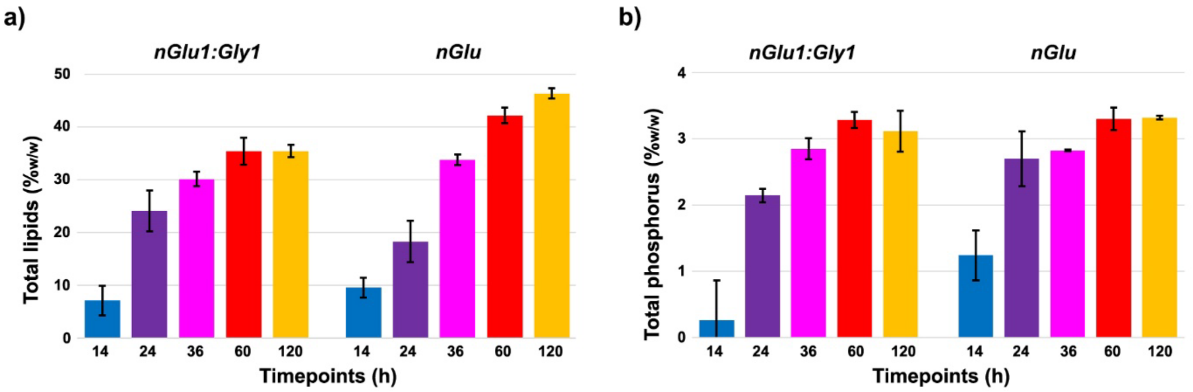

Figure S2. Estimates of total lipids and total phosphorus based on PLSR predictive models for FT-Raman spectra of bulk biomass of *Mucor circinelloides*. Colors represent different sampling timepoints: dark blue 14h, violet 24h, magenta 36h, red 60h and orange 120h. Samples are grouped according to the carbon source in the growth medium: ***nGlu1:Gly1*** glucose (^{12}C) 20 g/L and glycerol 20 g/L; ***nGlu*** glucose (^{12}C) 40 g/L.

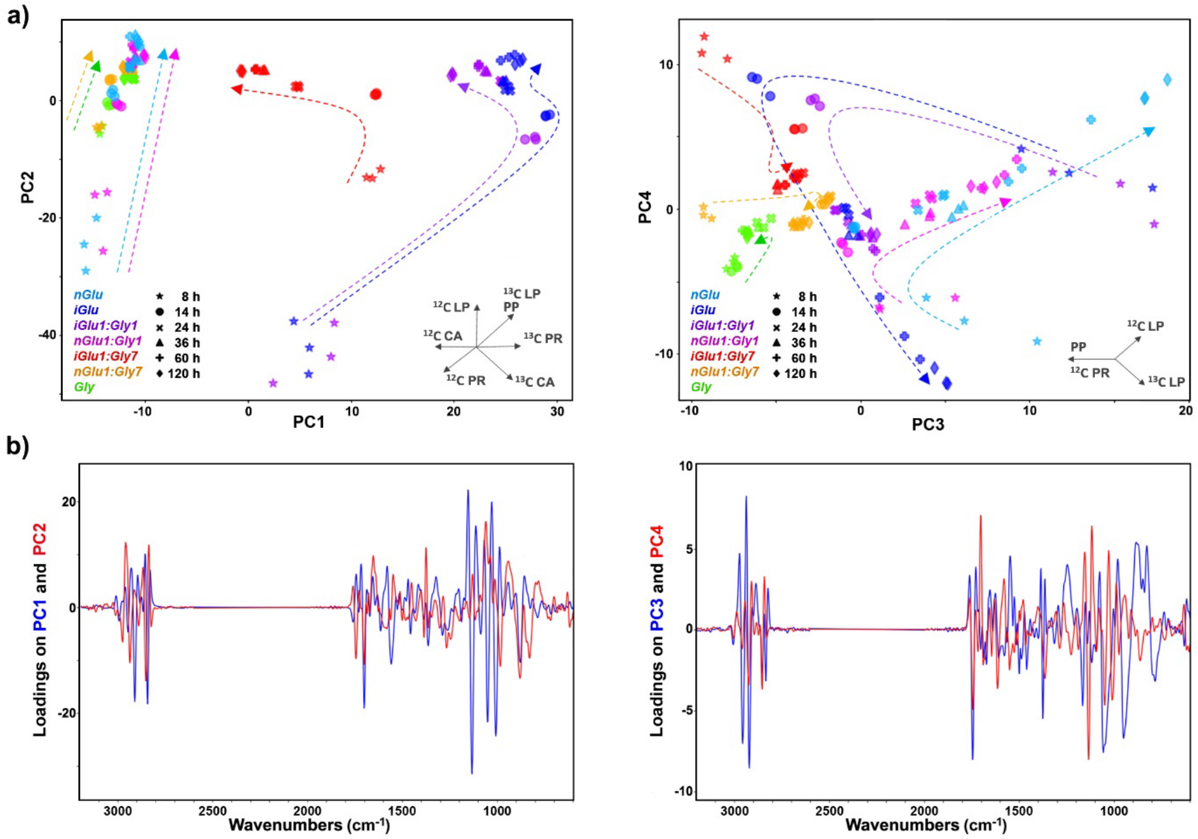

Figure S2. Multiblock consensus principal component analysis of HTIS-FTIR and HTS-FT-Raman spectroscopic data. (a) Score plots of CPCA HTS-FTIR individual blocks: The percent variances for the first five PCs are 50.60, 27.58, 8.54, 3.67 and 2.30. Vectors are approximating the increase in relative amount of the metabolites: lipids (LP), Proteins (PR), carbohydrates (CA), and polyphosphates (PP). (b) CPCA loading plots of HTS-FTIR individual blocks. Note that, as the spectral data for CPCA are in the second derivative form, high negative signal values correspond to high IR absorbance.

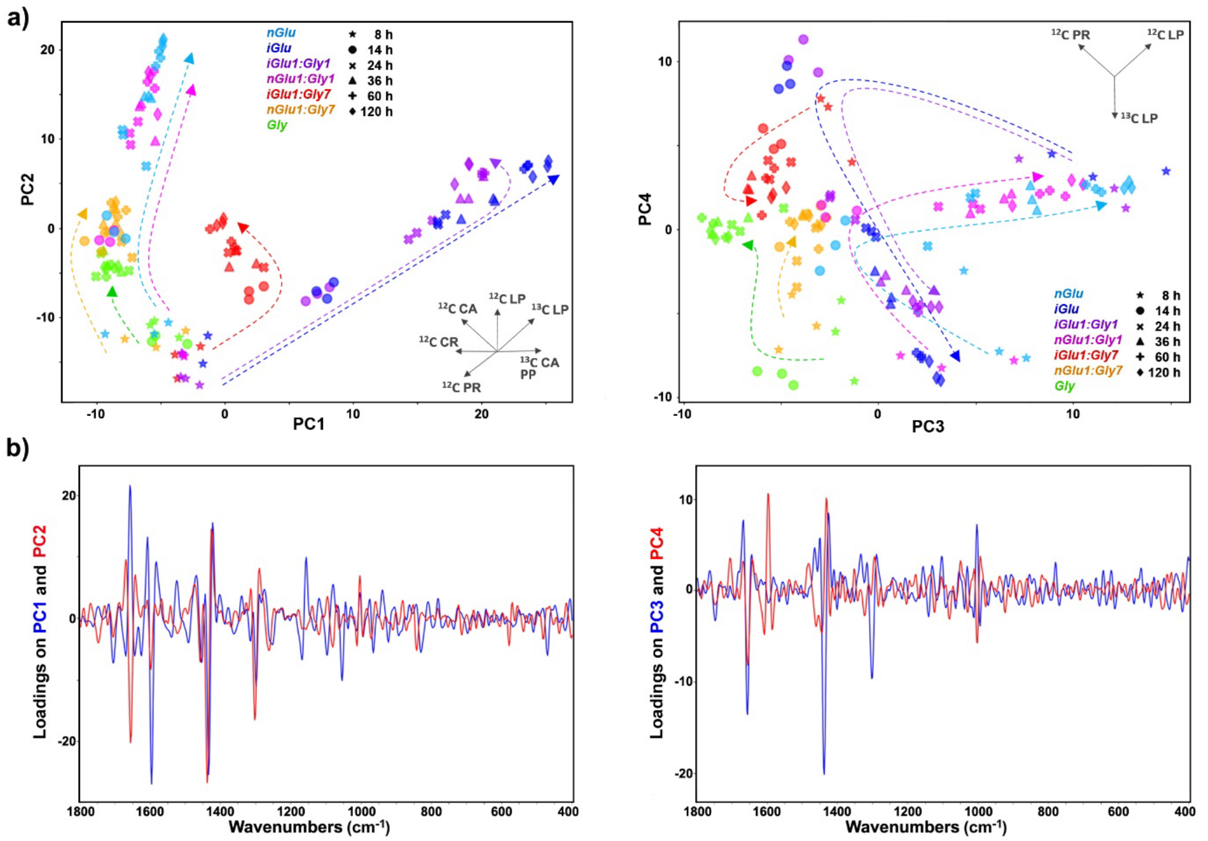

Figure S3. Multiblock consensus principal component analysis of HTIS-FTIR and HTS-FT-Raman spectroscopic data. (a) Score plots of CPCA HTS-FT-Raman individual blocks: The percent variances for the first five PCs are 27.69, 19.30, 9.54, 4.95 and 2.41. Vectors are approximating the increase in relative amount of the metabolites: lipids (LP), Proteins (PR), carbohydrates (CA), polyphosphates (PP), and carotenoids (CR). (b) CPCA loading plots of HTS-FT-Raman individual blocks. Note that, as the spectral data for CPCA are in the second derivative form, high negative signal values correspond to high Raman intensities.
